# Convex Analysis of Mixtures for Separating Non-negative Well-grounded Sources

**DOI:** 10.1038/srep38350

**Published:** 2016-12-06

**Authors:** Yitan Zhu, Niya Wang, David J. Miller, Yue Wang

**Affiliations:** 1 The Bradley Department of Electrical and Computer Engineering, Virginia Polytechnic Institute and State University, Arlington, VA 22203, USA; 2 The Program of Computational Genomics and Medicine, NorthShore University HealthSystem, Evanston, IL 60201, USA; 3 The Department of Electrical Engineering, Pennsylvania State University, University Park, PA 16802, USA

## Abstract

Blind Source Separation (BSS) is a powerful tool for analyzing composite data patterns in many areas, such as computational biology. We introduce a novel BSS method, Convex Analysis of Mixtures (CAM), for separating non-negative well-grounded sources, which learns the mixing matrix by identifying the lateral edges of the convex data scatter plot. We propose and prove a sufficient and necessary condition for identifying the mixing matrix through edge detection in the noise-free case, which enables CAM to identify the mixing matrix not only in the exact-determined and over-determined scenarios, but also in the under-determined scenario. We show the optimality of the edge detection strategy, even for cases where source well-groundedness is not strictly satisfied. The CAM algorithm integrates plug-in noise filtering using sector-based clustering, an efficient geometric convex analysis scheme, and stability-based model order selection. The superior performance of CAM against a panel of benchmark BSS techniques is demonstrated on numerically mixed gene expression data of ovarian cancer subtypes. We apply CAM to dissect dynamic contrast-enhanced magnetic resonance imaging data taken from breast tumors and time-course microarray gene expression data derived from *in-vivo* muscle regeneration in mice, both producing biologically plausible decomposition results.

Blind Source Separation (BSS) has proven to be a powerful and widely-applicable tool for the analysis of composite patterns in engineering and science, where both source patterns and mixing proportions are of interest but are unknown^1^,^2^,^3^,^4^. BSS is often described by a linear latent variable model **X** = **AS**, where **X** is the *M* × *N* observation data matrix containing *M* mixture signals with *N* data points, **A** is the unknown *M* × *K* mixing matrix, and **S** is the unknown *K* × *N* source data matrix containing *K* source signals with *N* dimensions. The fundamental objective of BSS is to estimate both the unknown mixing proportions and the source signals based only on the observed mixtures.

Many biomedical questions can be formulated as BSS problems, where the source signals are non-negative. For example, as we will show in one of our experiments, the dynamic contrast-enhanced magnetic resonance imaging (DCE-MRI) data of a tumor characterize a combination of distinct pharmacokinetics of different vascular compartments. The source signals in this case are the heterogeneous distributions of different vascular compartments within a tumor, which are non-negative and which usually contain Well-Grounded Points (WGPs), i.e. points with very high values in one source relative to all other sources^3^,^4^,^5^. Under the assumption of WGPs, column vectors of the mixing matrix **A** can be estimated by identifying WGPs located at the corners of the mixture observation scatter plot and, subsequently, the hidden source signals can be recovered. Based on the realization that the observed pattern across signal indices at each data point can be expressed as a non-negative combination of the column vectors of the mixing matrix^6^, we propose a Convex Analysis of Mixtures (CAM) method to estimate the mixing proportions by explicitly identifying WGPs at the lateral edges of the clustered observation scatter plot. CAM is theoretically supported by a series of newly proved identifiability and optimality theorems based on the noise-free case. A necessary and sufficient condition is discovered for identifying the mixing matrix through edge detection in non-negative well-grounded BSS problems, which serves as the foundation for CAM to identify the mixing matrix in the under-determined case, in addition to the exact-determined and over-determined cases. The optimality of the edge identification strategy is also proved for non-negative BSS problems, even when WGPs do not exist.

For applications on real-world noisy data, the CAM algorithm integrates a plug-in noise and outlier filtering scheme, an edge detection and geometric convex analysis algorithm, and a model selection scheme for applications on noisy real-world problems. We first design a sector-based clustering scheme, used to obtain an effective noise and outlier-reduced, clustered representation of the data. We then develop an efficient lateral edge detection and geometric convex analysis algorithm that identifies the WGP-associated clusters, whose center vectors are the estimates for the column vectors of the mixing matrix. The algorithm proceeds to estimate source signals by non-negative least-squares fitting of the latent variable model to the observation data, where the number of hidden sources is detected using a stability analysis scheme.

We demonstrate the principle and feasibility of the CAM approach on synthetic data and numerically mixed microarray gene expression profiles, and experimentally compare the accuracy of parameter estimates obtained using CAM to the most relevant alternative techniques. We then use the algorithm to dissect DCE-MRI data taken from breast tumors, identifying vascular compartments with distinct pharmacokinetics and revealing intratumor vascular heterogeneity. We also apply CAM to time-course gene expression data derived from *in-vivo* muscle regeneration in mice, observing biologically plausible dynamic patterns of relevant biological processes with distinct kinetics and phenotype-specific gene expression patterns. In Supplementary Information Section 1, we provide a brief review of existing BSS methods and discuss their relationship to CAM.

## CAM Theory

This section develops the theory of CAM for a noise-free scenario, including the model assumptions, identifiability, and optimality.

### Assumptions of the CAM Model

Considering the linear latent variable model **X** = **AS**, we can re-express the model in vector-matrix notation


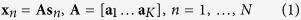


where **x**_*n*_, **a**_*k*_, and **s**_*n*_ are column vectors of matrices **X**, **A**, and **S**, respectively. Such a linear latent variable model is widely applicable to the analysis of many types of data, with the interpretation of the mixtures and underlying sources application-dependent. As a generic example for now, one can consider image unmixing, with *M* observed *N*-pixel images, each a mixture of *K* source images.

Our CAM model is developed based on the following assumptions.

(*A*1) (***Non-negative Sources***) Every element in **S** takes a non-negative value and **S** has full row rank.

(*A*2) (***Well-grounded Sources***) The source data matrix **S** contains at least one WGP on each of the *K* coordinate axes, i.e. 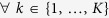, 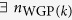 such that 
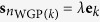
, *λ* > 0, where {**e**_*k*_} is the standard basis of *K*-dimensional real space.

(*A*3) (***Simplicial mixing matrix***) Every column vector in **A** is neither a non-negative nor a non-positive linear combination of other column vectors in **A**.

(*A*4) (***Full-rank mixing matrix***) **A** is of full column rank, i.e. rank (**A**) = *K*.

From (*A*1) and Equation 1, we have


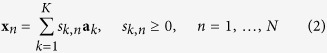


where *s*_*k,n*_ is the *k*th element of **s**_*n*_. When the source matrix **S** satisfies (*A*1) and (*A*2), i.e. it is a non-negative well-grounded BSS problem, (*A*3) is a necessary and sufficient condition for the mixing matrix **A** to be identified, as we will prove through a set of theorems later. (*A*4) is a sufficient condition for identifying the source matrix **S**, when (*A*1) and (*A*2) hold, and is widely used in many BSS problems^7^. Apparently, (*A*3) is a necessary but not a sufficient condition for (*A*4), in other words, (*A*3) is guaranteed to hold if (*A*4) is satisfied, but not vice versa. Also, importantly, (*A*3) can hold not only in the exact-determined and over-determined cases, but also in the under-determined case, where there are at least three mixtures, i.e. *M* ≥ 3. (*A*4) on the contrary can be satisfied only in the exact-determined and over-determined cases.

### Identifiability of the Mixing Matrix

We now discuss identifiability of the mixing matrix **A** under the aforementioned assumptions via the following definitions and theorems.

**Definition 1.** Given a matrix **B** composed by its set of column vectors {**B**} = {**b**_1_, …, **b**_*Q*_}, the convex cone determined by {**B**} is


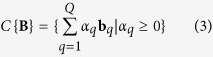


**Definition 2.** A non-zero vector **z** is a lateral edge of *C*{**B**}, if **z** ∈ *C*{**B**} (i.e. 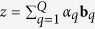, *α*_*q*_ ≥ 0) and **z** can only be expressed as a trivial combination of {**B**} (i.e. if *α*_*q*_ > 0 for some *q*, then **b**_*q*_ = *β*_*q*_**z**, *β*_*q*_ > 0).

**Figure 1:**
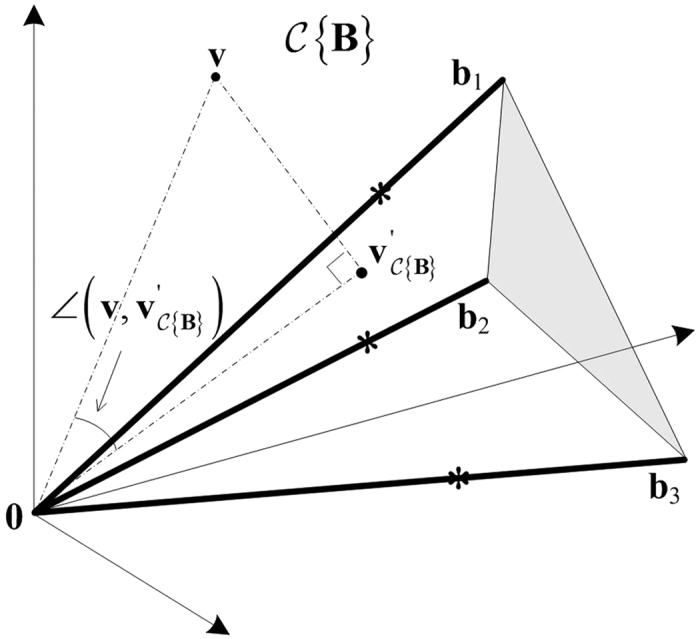
Illustration of a convex cone *C*{B} with three edges in three dimensional space. Lines with an arrow are the axes. Bold lines are edges **b**_1_, **b**_2_and **b**_3_. The cross-section of convex cone *C*{**B**} is a triangle, indicated by grey color. The star markers on the edges are well-grounded points. **v** is a point outside of *C*{**B**}. Its projection on *C*{**B**} is 
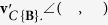
 denotes the angle between two input vectors.

See Fig. 1 for illustrations of a convex cone and its lateral edges. Because for edges only the vector direction is of interest, edges with the same vector direction but different lengths will be considered identical in the sequel. With the concept of convex cone, the model assumption (*A*3) can be formulated as


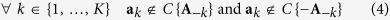


where **A**_−*k*_ is the matrix that results from removing the *k*th column from **A**.

**Lemma 1.** The lateral edges of the convex cone 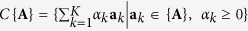 are the *K* (mixing matrix) column vectors **a**_1_, …, **a**_*K*_, if and only if (*A*3) holds.

**Lemma 2.** Suppose that (*A*1) and (*A*2) hold. Then, the convex cone defined by the observed data matrix, i.e. 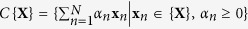, is identical to *C*{**A**}.

**Theorem 1. (Identifiability of the Mixing Matrix).** Suppose that (*A*1), (*A*2) hold. The mixing matrix column vectors **a**_1_, …, **a**_*K*_ can be determined by the lateral edges of *C*{**X**}, up to ambiguity of positive scaling and permutation, if and only if (*A*3) holds.

Please see Supplementary Information Sections 5 and 6 for the proofs of Lemma 1 and Lemma 2, respectively. Theorem 1 is a direct conclusion derived from Lemmas 1 and 2. It states that for separating non-negative well-grounded sources, (*A*3) is a sufficient and necessary condition for an edge detection solution uniquely identifying the mixing matrix **A** based on the observed data **X**. When WGPs exist, the lateral edges of cone *C*{**X**} are the mixing matrix column vectors **a**_1_, …, **a**_*K*_. That is, a WGP 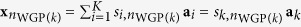 is a trivial combination of **a**_1_, …, **a**_*K*_, it is a lateral edge of cone *C*{**A**}, and since *C*{**A**} = *C*{**X**}, it is also a lateral edge of cone *C*{**X**}. This means that, in principle, under a noise-free scenario, we can directly recover **a**_1_, …, **a**_*K*_ by locating the lateral edges of *C*{**X**}, up to the ambiguity of positive scaling.

### Detectability of the Lateral Edges of Cone *C*{*X*}

From Theorem 1, we see that the key step to identify the mixing matrix is to detect the lateral edges of *C*{**X**}. Here we discuss the algorithmic principle and the optimality of an edge detection strategy via the following definition and theorems.

**Definition 3.** The projection of a point v onto the convex cone *C*{**B**} is


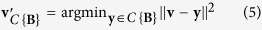


Obviously, if **v** ∈ *C*{**B**}, then 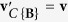 and 
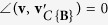
, where ∠(·,·) denotes the angle between two input vectors; if **v** ∉ *C*{**B**}, then 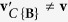 and 
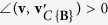
. We also define the angle between a non-zero vector and a zero vector to equal 180°, i.e. ∠(**v**, **0**) = 180°. See Fig. 1 for an illustration of projecting a data point onto a convex cone and the corresponding projection angle. The optimization problem in Equation 5 is a second order cone programming problem that can be solved by existing algorithms^8^.

**Theorem 2 (Property of lateral edges).** Suppose that (*A*1) and (*A*3) hold. Further, assume no two data vectors are in precisely the same direction. Let 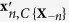 denote the projection of **x**_*n*_ onto cone *C*{**X**_−*n*_} where **X**_−*n*_ is the data matrix excluding **x**_*n*_. Then, **x**_*n*_ is a lateral edge of *C*{**X**}, if and only if 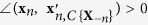.

Please see Supplementary Information Section 7 for the proof of Theorem 2. Theorem 2 immediately suggests a simple edge detection scheme to detect all lateral edges of *C*{**X**}. The scheme examines the data vectors one-by-one to check whether 
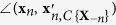
 is larger than 0, ∀*n*. If yes, **x**_*n*_ is a lateral edge of cone *C*{**X**}. Notice that this edge detection strategy does not require the existence of WGPs, but if WGPs do exist, the mixing matrix column vectors can be estimated using the lateral edges according to Theorem 1. Note that Theorem 2 and its associated edge detection strategy assumes each data vector **x**_*n*_ has a unique direction. This can be easily satisfied in practice by retaining in {**X**} only one data vector from each group of vectors that are positive scalings of each other (i.e., which lie in the same direction).

An important consideration for the present method is that it requires a WGP to exist for each of the underlying sources. While this is both a reasonable assumption in practice and serves to establish mathematical identifiability of the CAM model, nevertheless in some datasets, WGPs may not exist. It would be helpful to provide an accurate interpretation of the edge detection strategy in such non-ideal scenarios. Accordingly we show that, no matter whether WGPs exist or not, edge detection provides the *optimal solution* in the sense of capturing maximum source information. For each source, at least one lateral edge of *C*{**X**} achieves the *Maximum Source Dominance* (MSD) among all observed mixture data points and will be identified by edge detection. Specifically, we have:

**Theorem 3 (Source dominance optimality).** Suppose that (*A*1) and (*A*4) hold. For each source *k*, 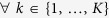, the edge detection strategy identifies at least one lateral edge, denoted by 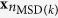, achieving the maximum source dominance in the sense of


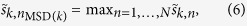


where 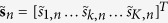, satisfying 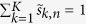, 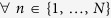 is the source vector of sample *n* following a normalization operation applied to the observed data matrix.

Please see Supplementary Information Section 8 for the proof of Theorem 3 and for the details of the normalization on the observed data matrix so that source vectors corresponding to different data points are comparable. Theorem 3 indicates that no matter whether WGPs are present or not, the edge detection strategy will identify the edges of *C*{**X**}, which are a group of observed mixture data points. And for each source, there is at least one detected edge that is the data point achieving the maximum dominance by this source among all observed data points, and it is the data point most similar to the corresponding mixing matrix column vector measured by source dominance, because the mixing matrix column vector can be considered purely dominated by one source with a source dominance value 1. So even when WGPs do not exist, edge detection still can identify the optimal estimates for the mixing matrix column vectors among all observed data points. WGPs, if they exit in the observed data, will become the lateral edges and be identified by edge detection.

### Summary of CAM Model Identifiability

From the above discussion, it has been established that, through edge detection, we can estimate the mixing matrix when the sources are well-grounded. Then, if (*A*4) is satisfied, the source data matrix **S** can further be recovered by the generalized inverse of **A**, which is **S** = (**A**^*T*^**A**)^−1^**A**^*T*^**X** under a noise-free scenario^7^. If (*A*4) is not satisfied and only (*A*3) is satisfied, **S** might not be recoverable.

We summarize the identifiability of the CAM model as follows:

(1) If (*A*1), (*A*2), and (*A*4) are satisfied, which can happen only in the exact-determined and over-determined cases, both **A** and **S** are identifiable.

(2) If (*A*1), (*A*2), and (*A*3) are satisfied (which can happen not only in the exact-determined and over-determined cases, but also in the under-determined case where there are at least three mixtures), the mixing matrix **A** and the number of sources are identifiable, while **S** cannot in general be uniquely determined.

## CAM Algorithm

So far, we have developed a mathematical CAM framework for separating non-negative well-grounded sources under an ideal noise-free situation. In this section, we develop a practical CAM algorithm that is based on this theoretical framework but which also robustly addresses the realistic scenario where there may be both noise and outliers present. This algorithm consists of data preprocessing, sector-based clustering, convex analysis of mixtures, stability analysis, and source pattern recovery. We first summarize the steps of the CAM algorithm and then explain each of these steps in the following sub-sections.

### CAM Algorithm

(1) Data preprocessing to normalize data and remove data points with small vector norms that potentially have low local SNR.

(2) Sector-based clustering on the scatter plot to get a noise-reduced representation of the data.

(3) Convex analysis of mixtures for estimating the mixing matrix, including (i) edge detection based on sector central rays to form a candidate pool of estimates for the mixing matrix column vectors and (ii) minimization of model fitting error to produce an estimate for the mixing matrix with a given source number.

(4) Determination of source number by stability analysis, which repeats steps (2) and (3) for different source numbers based on random partitions of the data to calculate the normalized model instability of each candidate source number. The best source number is selected as the one with the smallest instability.

### Data Preprocessing

Our algorithm begins with two data preprocessing steps. First, we scale the observed mixtures to have unit sums and assume the underlying sources also have unit sums as done in the literature^5^, i.e. after scaling, 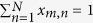, ∀*m* = 1, …, *M*, and 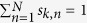, ∀*k* = 1, …, *K*. Note that this scaling makes each row of **A** have unit sum so that the mixing matrix elements provide the mixing proportions, i.e. 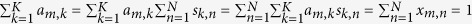, 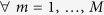, where *a*_*m,k*_ is the *m*th element of **a**_*k*_. Second, consider the following noisy linear latent variable model


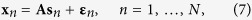


where **ε**_*n*_ is the additive noise on sample *n* and is independent of **s**_*n*_. We assume that 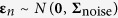 and define the Signal-to-Noise Ratio (SNR) of the whole dataset as


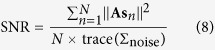


Since the expected noise level for all data points is the same, data points with small vector norms are expected to have a lower local SNR, which could have a negative impact on subsequent analysis^3^, so the second step of data preprocessing is to exclude these small-norm points.

### Noise or Outlier Removal by Sector-based Clustering

**Figure 2:**
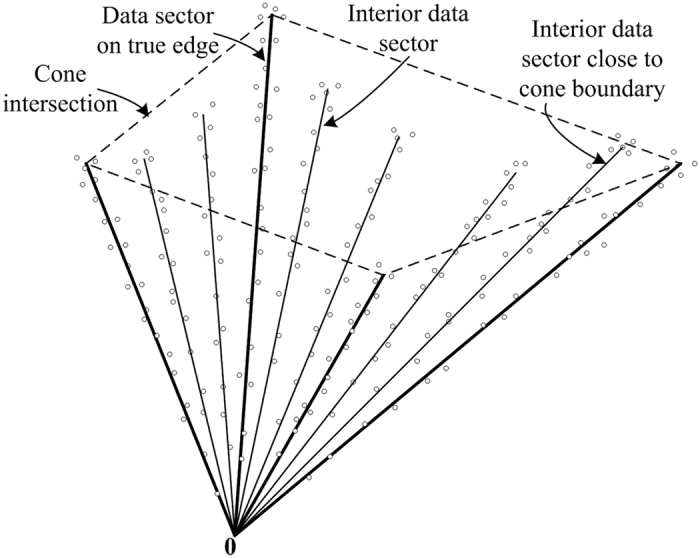
Illustration of sector-based clustering in a three-dimensional scatter plot. Four sources (*K* = 4) are mixed to form three mixtures (*M* = 3). Small circles are data points. After clustering, each data sector is represented by a sector central ray (solid lines). Four data sectors are on (or close to) the true edges of cone *C*{**X**}, with their sector central rays indicated by bold lines. The quadrilateral formed by the dashed lines indicate the intersection of the cone.

The purpose of sector-based clustering on the preprocessed data points is two-fold: 1) data clustering has proven to be an effective tool for reducing the impact of noise and outliers on model learning^3^,^9^; and 2) aggregation of data points into a (smaller) number of clusters improves the computational efficiency of the subsequent convex analysis of mixtures by reducing the number of tests performed for identifying lateral edges. After sector-based clustering, each data sector (cluster) is represented by a ray starting from the origin, which is called a sector central ray. Please see Fig. 2 for illustration of sector-based clustering.

**Definition 4.** The sector central ray **r**_*j*_ of the *j*th data sector is the ray starting from the origin that minimizes the sum of the squared distances to all the data points in the *j*th data sector.

The distance between a data point and a ray is the minimum distance between the data point and any point on the ray. Sector-based clustering groups data points into sectors (each with its own central ray) so that data points within a sector have more similar orientations (evaluated by their angles made with the central ray) compared to data points in other sectors. Assuming a sufficient number of sectors are used to model the data, we logically impose ∠(**x**_*n*_, **r**_*j*_) ≤ 90°, ∀**x**_*n*_ ∈ *ψ*_*j*_, ∀*j* ∈ {1, …, *J*}, where *J* is the number of data sectors and *ψ*_*j*_ denotes the *j*th data sector. Since only the vector direction is of importance, the sector central rays are confined to have unit norm, i.e. 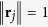, 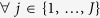. Based on Definition 4, the sector central ray is mathematically defined as


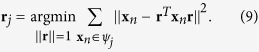


By expanding the square in the summation and simplifying, we can show that


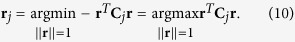


where 
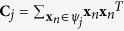
 is (the sample-based estimate of) the autocorrelation matrix of data vectors in *ψ*_*j*_. The solution of Equation 10 is the principal eigenvector of **C**_*j*_.

### Sector-based Data Clustering Algorithm

(1) Randomly initialize each of the *J* sector central rays **r**_1_, …, **r**_*J*_ to one of the observation data points **x**_1_, …, **x**_*N*_ and unit-normalize these vectors.

(2) Partition the observed data points into *J* data sectors by assigning each data point to its nearest sector based on the distance between the data vector **x**_*n*_ and the sector central ray **r**, calculated by 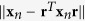.

(3) Update the *J* sector central rays **r**_1_, …, **r**_*J*_ by finding the principal eigenvector of each of the sample-based correlation matrices **C**_*j*_, *j* = 1, …, *J*, determined by the data partition in step (2).

(4) Terminate if there is no change in the total clustering distortion shown in Equation 11, from the previous to the current iteration; otherwise, go to step (2).

The sector-based clustering algorithm monotonically descends in the clustering distortion


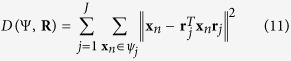


where **R** = [**r**_1_ … **r**_*J*_] is the matrix composed of sector central rays and Ψ is the partition of data points into *J* data sectors. It also terminates in a finite number of iterations at a fixed point solution that is a local minimum of Equation 11, which can be proved following the standard convergence proof of the generalized Lloyd algorithm^10^,^11^. The computational complexity of this algorithm is dominated by the partitioning step, whose complexity is O(*JMNI*), where *I* is the number of algorithm iterations. Random initialization of the sector central rays can affect the local optimum to which the algorithm converges; thus, in practice, the algorithm is usually run multiple times, with the sector partition with the minimum clustering distortion chosen as the final outcome.

### Convex Analysis of Mixtures

At this juncture, having performed sector-based clustering, we have **R** = [**r**_**1**_ … **r**_*J*_] as a noise/outlier mitigated representation of the data matrix **X**. Accordingly, supported by Theorem 1, which says that in the noise-free case, the columns of **A** are the lateral edges of *C*{**X**}, it is reasonable, in the noise-mitigated case, to estimate the columns of **A** based on the lateral edges of the cone *C*{**R**}. CAM uses the following algorithm specifically designed based on Theorem 2 to detect the lateral edges of cone *C*{**R**}.

### Cone Lateral Edge Detection Algorithm

(1) Set **R**_edge_ = **R**, *j* = 1, and *τ* = 0.001 (or another small positive value); Set J* = *J.*

(2) Determine projection image 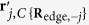 by projecting **r**_*j*_ onto cone *C*{**R**_edge,−*j*_}, where **R**_edge,−*j*_ is the matrix resulting from removing the *j*th column from **R**_edge_.

(3) If 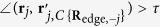, *j* = *j* + 1; otherwise, remove **r**_*j*_, i.e. the *j*th column, from **R**_edge_ and J* = J* − 1.

(4) If *j* > J*, end the algorithm; otherwise, go to step (2).

The worst-case computational complexity of the cone lateral edge detection algorithm is *O(J*^3^*M*). After applying the algorithm, the J* column vectors in **R**_edge_ are the detected edges. The detected edge number has some dependence on the sector-based clustering solution, including the chosen number of sectors, *J*. Clearly, one must choose *J* > *K*. In practice, to ensure this, one may choose *J* fairly large, in which case there are usually more than *K* detected edges. Regardless, the detected edges are good candidates from which to select a subset as the estimates of **a**_1_, …, **a**_*K*_.

To identify good, refined estimates of **a**_1_, …, **a**_*K*_ from this candidate pool, a combinatorial search based on a model fitting error criterion can be performed to identify the most promising *K* lateral edges. Specifically, let 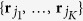 be any size-*K* subset of {**R**_edge_}. The *K* lateral edges with sector indices 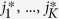 that minimize a model fitting error are chosen, as follows:


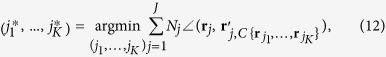


where 
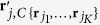
 is the projection of **r**_*j*_ onto cone 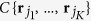 and *N*_*j*_ is the number of data points in sector *j*. Because the angles between the “interior” sector central rays, i.e. sector central rays confined within 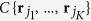, and their projections on 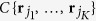 are all 0, the model fitting error is a weighted sum of the angles between the “exterior” sector central rays and their projections, and the weights are the data sector population sizes. Because this model fitting error is monotonically decreasing as the edge set under consideration is enlarged, the search for the best *K* lateral edges can be accelerated by using the branch and bound search algorithm^12^, which guarantees finding the edge set minimizing the model fitting error without the need for exhaustive search. The average complexity of branch and bound search is no larger than 
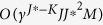
, where *γ* > 1 is a constant that is problem-dependent^13^.

The edge set minimizing the model fitting error forms the estimate of the mixing matrix, which we denote by 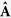. We then project all the mixture data vectors (including the small-norm data vectors removed in data preprocessing) onto the cone 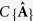 and compose these projected vectors into a matrix, 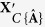. This projection step ensures that our estimates for the sources will be non-negative and also helps to suppress noise. If 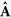 has full column rank, the estimates of sources are calculated via the generalized inverse of 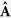, i.e. 
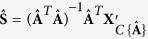
. Because 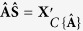, which is the projection of the original mixture data matrix **X** onto the cone 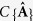, it can be shown that 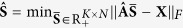, where 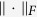 denotes the Frobenius norm of a matrix and 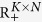 denotes the set of *K* by *N* non-negative matrices. Thus, 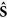 is actually a non-negative least squares estimate.

### Detection of Source Number by Stability Analysis

One important CAM issue is detection of the structural parameter *K* (the number of underlying sources), often called model selection. This is indeed particularly critical in real-world applications where the true structure of the latent variable model may be unknown *a priori*. We propose to use a stability analysis scheme to guide model selection, based on a carefully designed model instability index.

Similar to the rationale in determining the number of clusters in data clustering using stability analysis^14^, the basic principle is that, if *K* is too large, some extracted sources will simply model random noise patterns; on the other hand, if *K* is too small, some extracted sources will be arbitrary combinations of true sources; both scenarios produce unstable models. Stability analysis assesses the model instability indices associated with different values of *K*, calculated based on a large number of 2-fold cross-validations, and selects the model order with lowest model instability. In each cross-validation trial *l* ∈ {1, …, *L*}, the preprocessed observation data are randomly divided into two folds (indexed by *l*_1_ and *l*_2_) of equal size; then, CAM is applied on both folds and produces two independent estimates of the mixing matrix, denoted as 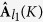 and 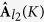, respectively, for 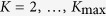, where *K*_max_ is the maximum source number under consideration. We then define the Normalized Model Instability (NMI) index as


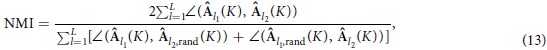


where 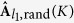 and 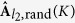 are estimates of the mixing matrix formed by randomly selecting *K* sector central rays from the sector-based clustering result obtained on data folds 1 and 2 in the *l*th cross-validation, respectively, and where ∠(·,·) here denotes the minimum average angle between the column vectors of two input matrices. To explicate this averaging, let the two input matrices be **U** = [**u**_1_ … **u**_*K*_] and **W** = [**w**_1_ … **w**_*K*_]. ∠(**U**, **W**) is calculated as


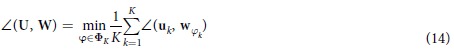


where **Φ**_*K*_ is the set including all permutations of {1, …, *K*} and *φ*_*k*_ is the *k*th element in a permutation **φ**. Since the association between column vectors in **U** and **W** is not known, we need to search through all possible associations to find the optimal one. Using the Hungarian method, the complexity of this search is *O(K*^3^)^15^. The definition in Equation 13 produces an NMI index that is easy to interpret, and the “normalization” automatically adjusts the NMI index for comparison across different model orders as adopted by other works^14^.

## Results

### Demonstration of CAM Performance on Synthetic Data and Numerically Mixed Data

To illustrate CAM, we first consider a simulated data set consisting of *N* = 1600 data points. Half of the source vectors are drawn from a three-dimensional exponential distribution with independent variables to ensure the existence of approximate WGPs. The other half are first drawn from a three-dimensional Gaussian distribution with correlated variables to ensure source dependence and then absolute values are taken to force source non-negativity. The mixing matrix, source mean vectors, and covariance matrix are given as


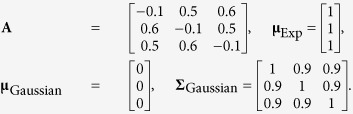


The additive noise is drawn from a Gaussian distribution with


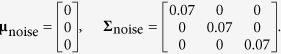


The structure of this data set has been chosen in order to illustrate the noisy and strongly correlated nature of many real data sets. The exponential distribution with independent variables has a heavy density in the vicinity of the origin for each dimension, which gives a good chance for WGPs or near-WGPs to exist. The highly correlated Gaussian distribution makes the simulated sources correlated with each other. The dataset has an SNR of 12.4 dB, calculated by Equation 8.

After data preprocessing, we kept the 800 data points whose vector norms are largest and performed sector-based clustering on these data points 20 times with *J* = 30, selecting the best clustering outcome measured by the total clustering distortion given in Equation 11. On the sector central rays obtained from the best clustering outcome, we performed the cone lateral edge detection algorithm and then identified the three edges that minimized the model fitting error according to Equation 12 to form the estimate of the mixing matrix. The sources were recovered using the mixing matrix estimate accordingly.

We utilized the minimum average angle between the estimated mixing matrix column vectors and the true mixing matrix column vectors to evaluate how accurately the mixing matrix was recovered. We used the average correlation coefficient between the estimated sources and the true sources to measure the recovery accuracy of sources. See Supplementary Information Section 2 for the formulas defining the recovery accuracy of the mixing matrix and the recovery accuracy of the sources. Both accuracy measures are between 0 and 1, with 1 indicating perfect estimation.

**Figure 3:**
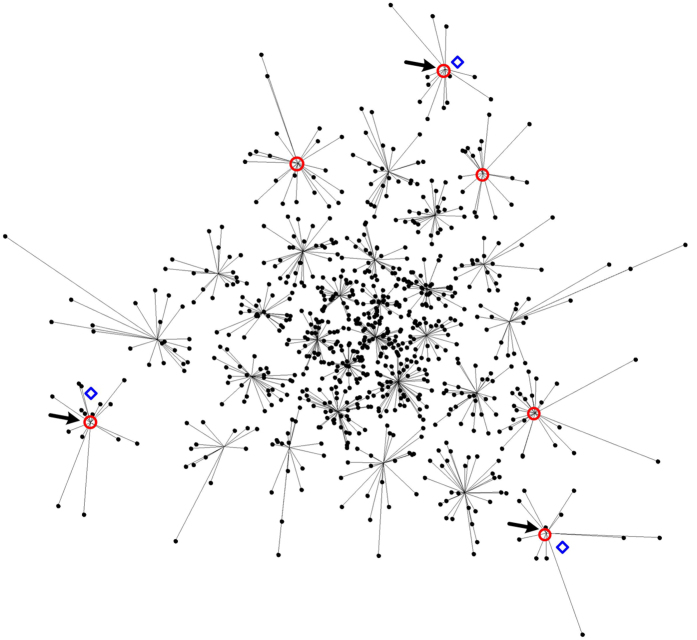
Perspective projection of the 800 large-norm data points in the simulation dataset onto the 2-D intersection of the convex cone formed by the data points. Perspective projection performs simple positive scaling of data points to make every data point have unit element sum. Black dots are data points. Each data point is connected to its sector central ray by a line. Red circles indicate the edges detected by applying the lateral edge detection algorithm on the sector central rays. Blue diamond markers indicate the positions of true mixing matrix column vectors. The three edges that minimize the model fitting error among all three-edge sets are indicated by arrows.

Figure 3 shows the 800 large-norm data points, the best clustering outcome from 20 runs, the edge detection result, and the estimates of the mixing matrix column vectors. We applied stability analysis with 30 cross-validations, and obtained NMI indices that show a minimum value at *K* = 3 (see Table 1 for NMI indices of different model orders), which agrees with the ground truth. With *K* = 3, the resulting recovery accuracies of the mixing matrix and sources were 0.9826 and 0.9171, respectively. The power of the CAM approach is supported here as both the mixing matrix and hidden sources are well recovered and the number of hidden sources is correctly identified.

We compared the performance of CAM with eight most relevant methods (see Supplementary Information Section 3), including non-negative Independent Component Analysis (nICA)^16^, Statistical Non-negative Independent Component Analysis (SNICA)^17^, Non-negative Matrix Factorization (NMF)^1^, Sparse Non-negative Matrix Factorization (SNMF)^18^, N-finder algorithm (N-FINDR)^19^, Vertex Component Analysis (VCA)^20^, Convex Analysis of Mixtures of Non-negative Sources (CAMNS)^5^, and nonnegative Least-correlated Component Analysis (nLCA)^4^, which cover many different BSS algorithm categories, such as deterministic methods vs. probabilistic methods, methods with the well-grounded source assumption vs. methods without it, and methods assuming sources are independent or uncorrelated vs. methods that assume sources may be correlated. The comparison was conducted on numerical mixtures of gene expression profiles of four ovarian cancer subtypes generated by Schwartz *et al*.^21^ Experimental methods used to produce the gene expressions were carried out in accordance with the approved guidelines; analysis of all human ovarian cancer tissues was approved by the University of Michigan’s Institutional Review Board (IRB-MED no. 1999-0428); and informed consent was obtained for all human participants^21^. We added noise to the mixture data, and tested the methods over a range of SNR (19 dB~34 dB) using multiple simulation datasets. CAM always outperformed all eight peer methods in both the exact-determined scenario (*M* = *K* = 4) and the over-determined scenario (*M* = 6 > *K* = 4) for recovering the mixing matrix and sources (Fig. S1). When SNR was 25 dB or higher, CAM always correctly detected the number of sources (Fig. S2). We tested CAM’s ability to recover the mixing matrix in the under-determined scenario (*M* = 3 < *K* = 4). When the source number was given, CAM recovered the mixing matrix reasonably well over the tested SNR range (Fig. S3a). When the SNR level was higher than 25 dB, CAM showed a source number detection accuracy higher than 80% (Fig. S3b). We also recorded the execution times of the competing methods in Table S1 for a comparison of their computational complexities.

### Analysis of Breast Cancer DCE-MRI Data

As an example of using CAM for real-world application, we considered DCE-MRI data from breast cancer to evaluate tumor vasculature patterns^3^,^22^. The data were collected under a protocol approved by the National Institutes of Health (NIH) institutional review board after informed consent was obtained from the study participant. The images were generated according to approved guidelines. The data include MRI images of breast tumors taken at sequential time points after the injection of molecular contrast agent into the blood. Due to intratumor heterogeneity and limited imaging resolution, the concentrations of the contrast agent at many image pixels often represent a mixture of more than one vascular compartment, each with distinct and characteristic perfusion and permeability. The existence of near-pure compartment pixels allows us to use CAM to identify distinct vascular compartments and their spatial distributions within a tumor.

**Figure 4:**
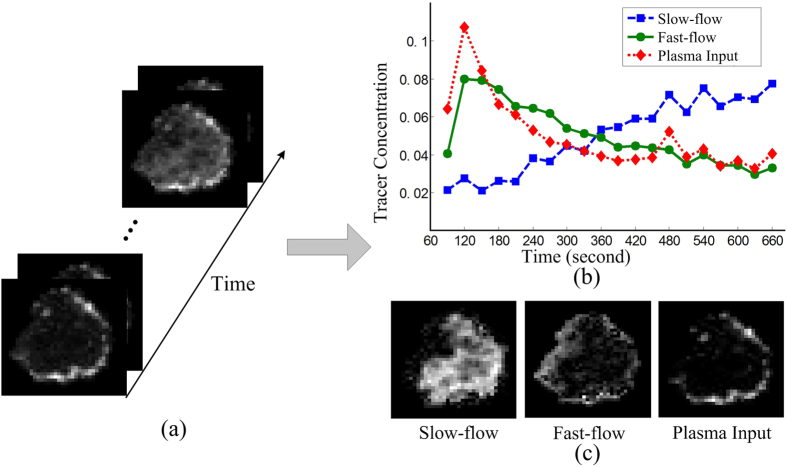
CAM analysis result on breast cancer DCE-MRI data. (**a**) MRI images of a breast tumor taken at sequential time points after the injection of molecular contrast agent into blood. (**b**) Tracer concentration changes of the three identified compartments over time. (**c**) Recovered source images of the three compartments.

The DCE-MRI dataset includes *M* = 20 image frames of a breast tumor (see Fig. 4a) taken every 30 seconds, starting from 90 seconds after injection of the molecular contrast agent. Each image contains 50 × 50 = 2500 pixels, and after masking out the non-tumor region, the resulting image contains *N* = 715 pixels for CAM analysis. Noise filtering removed 30% of the pixels whose vector norms were small. The sector-based clustering chose the best clustering outcome in 20 independent runs, with cluster number *J* = 30. We performed stability analysis via 30 cross-validations, which suggested the compartment number *K* = 3, as summarized in Table 1.

CAM analysis indicates three compartments, i.e. fast-flow, slow-flow, and plasma input^23^, characterized by their pharmacokinetics patterns. Figure 4b shows the dynamic changes of tracer concentration of the three compartments, which are the column vectors in the recovered mixing matrix 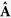. Each column vector was scaled to have a unit sum for drawing Fig. 4b. Figure 4c shows the spatial distributions of the identified compartments, which correspond to the recovered sources 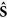.

The fast-flow compartment has a fast tracer clearance rate (see Fig. 4b) and dominates the peripheral “rim” of the tumor (see Fig. 4c). The slow-flow compartment shows very slow tracer kinetics (see Fig. 4b) and dominates the inner “core” of the tumor (see Fig. 4c). The identification of fast-flow and slow-flow pools is plausibly consistent with previously reported intratumor heterogeneity^22^,^24^. The defective endothelial barrier function of tumor vessels results in spatially heterogeneous high microvascular permeability to macromolecules^22^,^24^. It has been reported that the peripheral “rim” of advanced breast tumors often have active angiogenesis that is essential to tumor development^24^. This rapidly proliferating neovasculature is often abnormal, and forms leaky and chaotic vessels, giving rise to a rapid tracer uptake and washout pattern, forming the fast-flow pool^22^. On the other hand, the inner “core” of the tumor has significantly lower blood flow and oxygen concentration, forming the slow-flow pool with much slower tracer accumulation and washout, because the tumor growth in peripheral “rim” region requires a large portion of its blood supply and also neovessel maturation^22^.

### Analysis of Muscle Regeneration Time-Course Gene Expressions

We applied CAM to dissect a time-course gene expression dataset obtained from a mouse skeletal muscle regeneration process^25^ (GEO accession no. GSE469). Skeletal muscle regeneration is a highly synchronized process involving the activation of various cellular processes. Cells grow in dynamically evolving subpopulations, yet the dynamics and proportions of cell subpopulations often go unmeasured on the basis of their mRNA expression patterns^26^. Within a mixed population of cells, one might expect distinct cell types to exhibit some distinct patterns of gene expression, and the measured mRNA levels in the mixed cell population represent a weighted average of these hidden biological processes, where the weights are cell proportions involved in different biological processes. Here, we ask whether it is possible to deconvolve the gene expression data from a mixed cell population to discern the proportions of different cell types, by treating specific mRNA patterns as cell-type specific markers^26^.

The time-course muscle regeneration gene expression data were acquired at *M* = 27 successive time points using microarrays after the injection of cardiotoxin into the mouse muscle, which damages the muscle tissue and induces staged muscle regeneration^25^. Standard preprocessing suggested *N* = 7570 reliably expressed genes for subsequent CAM analysis^25^. Noise filtering removed 40% of the genes whose vector norms were small. The sector-based clustering chose the best clustering outcome in 20 independent runs, with cluster number *J* = 30. We performed stability analysis via 30 cross-validations, which suggested *K* = 4 as the number of potentially distinct sources associated with underlying active biological processes, as summarized in Table 1.

**Figure 5:**
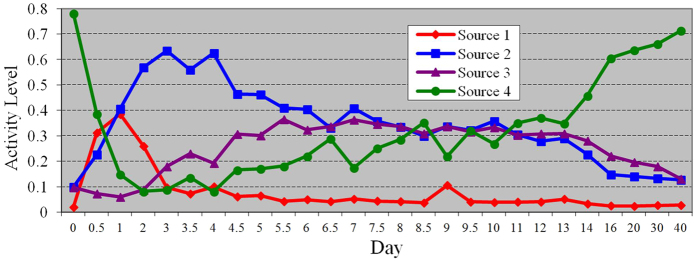
Time activity curves of the four sources detected on the 27 time-point skeletal muscle regeneration gene expression dataset.

Figure 5 displays the source-specific time activity curves (the column vectors of the estimated mixing matrix) that represent the proportions of cell subpopulations associated with the 4 underlying putative biological processes at each time point. For each of the identified sources, we selected 200 source-specific genes (near-WGPs) that maximize 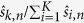, ∀*k* = 1, …, 4, to define source-specific distinct patterns^27^. We input the four source-specific gene groups into Ingenuity Pathway Analysis (IPA), a comprehensive database of gene annotations and functions that performs Fisher’s exact test to assess the association of a given gene set with known biological functions, with *p*-values indicating the significance level. Functional analysis by IPA consistently suggests the biological plausibility of all four biological processes revealed by CAM.

Specifically, IPA suggests that source 1 is associated with inflammation, connective tissue disorders, skeletal and muscular disorders, and immune response, with *p*-values of 6.77E-39, 9.02E-35, 9.02E-35, and 9.62E-32, respectively. The corresponding genes are heavily involved in the necrosis of damaged muscle tissue and the activation of an inflammatory response. In Fig. 5, it can be seen that source 1 activates immediately after muscle damage and then diminishes quickly, reflecting the fact that necrosis and inflammatory response constitute the first transient phase of muscle regeneration^28^. IPA suggests that source 2 is associated with three biological functions, i.e. (1) cell cycle, (2) DNA replication, recombination, and repair, and 3) cellular growth and proliferation, with *p*-values of 7.07E-25, 3.77E-17, and 2.10E-8, respectively. The associated genes are actively involved in myogenic cell proliferation to prepare sufficient myoblasts for later differentiation. The source 2 activity reaches its peak(s) from day 2 to day 4 as biologically expected (see Fig. 5)^28^. IPA suggests that source 3 is associated with tissue development, skeletal and muscular system development, cell to cell signaling and interaction, and connective tissue development and function, with *p*-values of 9.09E-16, 4.91E-11, 2.33E-08, and 4.35E-07, respectively. The corresponding genes are expected to facilitate the differentiation of myoblast into mononucleated myocyte and the fusion of myocytes to form multinucleated myofibers. As expected, in Fig. 5 the source 3 activity goes up after sufficient myoblasts are produced by the activity of source 2, keeps at a high level from day 5 to day 13, and then goes down. Such a trend is consistent with the widely observed fact that muscle regeneration is accomplished in approximately two weeks^28^. IPA suggests that source 4 is associated with skeletal muscular system function and tissue morphology, with a *p*-value of 3.49E-10. The corresponding genes are typically active in normal muscle cells, whose activity drops dramatically after muscle is damaged and gradually recovers until it finally reaches a similar level of original muscular activity as at day 0 (see Fig. 5).

## Conclusion and Discussion

We have presented a novel approach to separate non-negative well-grounded sources from observed mixtures, which is geometrically principled and which, as illustrated by the real data example, can be very effective at revealing hidden sources within data. It is worth noting that there are four novel features/contributions associated with our work. First, we show both feasibility and optimality of the CAM model via newly proved theorems for the noise-free case. We prove for the first time a necessary and sufficient condition (i.e. assumption (*A*3)) for identifying the mixing matrix in non-negative well-grounded BSS problems through edge detection. We also show the optimality of the edge detection strategy that identifies the data points with maximum source dominance, even when WGPs do not exist. Second, we develop the practical, noise-tolerant CAM algorithm, a novel BSS method that integrates an effective noise and outlier removal scheme based on sector-based clustering, an efficient lateral edge detection method on the clustered data scatter plot, and a model order selection scheme based on stability analysis. Third, the proposed CAM method can be uniformly applied to the exact-determined, over-determined, and under-determined cases for identifying the mixing matrix, while most existing methods can work in only one or two of the situations. Fourth, we applied CAM to analyze breast cancer DCE-MRI data and *in vivo* mouse muscle regeneration gene expression data, and obtained biomedically plausible results. On the breast cancer DCE-MRI data, CAM discovered intratumor vascular heterogeneity showing distinct pharmacokinetics. On the mouse gene expression data, CAM discovered the dynamic signals of molecular biological processes regulating the regeneration of skeletal muscle.

Over the past twenty years, a variety of BSS techniques have been continuously reported and tested on synthetic and real data^7^,^29^,^30^,^31^,^32^,^33^,^34^,^35^,^36^. We provide a brief review of existing BSS methods in Supplementary Information Section 1. Some of the BSS methods also exploit the source well-groundedness assumption as CAM does. But CAM has key differences and advantages over these methods, such as the novel features summarized above and that CAM does not require the mixing matrix to be non-negative nor fully column ranked. We provide more discussion on the relationship between CAM and other methods in Supplementary Information Section 1. The proposed CAM method is largely a deterministic approach. There is usually a connection between deterministic BSS methods and probabilistic BSS methods^37^. We are currently investigating a probabilistic CAM model that combines geometric convex analysis with probabilistic modeling. Within a probabilistic modelling framework, information-theoretic criteria, such as minimum description length^3^, can be used for model selection to determine the source number.

The CAM algorithm uses three hyperparameters, including *τ* in the cone lateral edge detection algorithm, the sector number *J* in sector-based clustering, and the percentage of the small-norm data points to be excluded for estimating the mixing matrix. All analysis results in our study were obtained with *τ* = 0.001. The value of *τ* usually does not affect the analysis result, so long as it is sufficiently small. The edge detection is performed based on sector central rays, which usually have quite different vector directions from each other. A sector central ray identified as an edge most likely has a significant deviation (much larger than *τ*) from the cone formed by other sector central rays. In the performance comparison experiment, we examined the performance of CAM with *J* equal to 20 and 30, which are labeled as CAM-20S and CAM-30S, respectively (see Section 3 of the Supplementary Information). We found both CAM-20S and CAM-30S outperform the competing methods over the tested SNR range, which indicates that application of CAM with a flexible choice of *J* yields good performance. Moreover, there are methods for identifying an optimal cluster/sector number, such as the stability-based cluster number detection method proposed by Lange *et al*.^14^, which can be used for determining a suitable sector number. For simplicity, we fixed *J* at 30 when analyzing the real datasets in our study. In the data preprocessing step of CAM, a portion of the data points whose norms are small are excluded for the estimation of the mixing matrix. Section 4 of the Supplementary Information evaluates how sensitive the analysis result of CAM is to the change in the percentage of data points that are excluded. On both the breast cancer DCE-MRI data and the skeletal muscle regeneration gene expression data, the CAM outputs, including the estimated mixing matrix and sources, are stable when the percentage of excluded data points changes over a relatively large range, i.e. 30%~50% (see Section 4 of the Supplementary Information). This preprocessing step is designed to exclude the small-norm data points with very low local SNR that may jeopardize the CAM analysis. Excluding a sufficient portion of the data points, such as 30%, to avoid such low SNR data is usually a good starting point for practical use of CAM. Several analysis trials with different percentages of removed data points, such as 40% and 50%, can then be performed. These analyses may generate similar results, as we have observed in our sensitivity study. If so, this supports the use of the removal percentage that was initially chosen. Also, for many exploratory studies, there may exist some domain knowledge (although not complete) that can help indicate which analysis result is more interpretable and interesting.

In the analysis of muscle regeneration data, we selected 200 genes specific to each source for the pathway enrichment analysis. The considerations here are that many genetic pathways include no more than 200 genes and, thus, including too many genes in the enrichment analysis may yield enlarged p-values associated with pathways that are actually significantly enriched in the genes that are most specific to a source. As general guidance on using CAM to analyze gene expression data for biological study, 200 or a comparable number would be a good starting number for selecting source-specific genes in the pathway enrichment analysis.

An open-source platform-independent CAM software package in R-Java is available at: http://mloss.org/software/view/437/.

## Additional Information

**How to cite this article**: Zhu, Y. *et al*. Convex Analysis of Mixtures for Separating Non-negative Well-grounded Sources. *Sci. Rep.* **6**, 38350; doi: 10.1038/srep38350 (2016).

**Publisher's note:** Springer Nature remains neutral with regard to jurisdictional claims in published maps and institutional affiliations.

## Supplementary Material

Supplementary Information

## Figures and Tables

**Table 1:** NMI indices associated with different source numbers obtained when applying CAM on the datasets.

Source Number		2	3	4	5	6	7	8	9
NMI Index	Synthetic data	0.79	**0.21**	0.54	0.60	0.65			
	DCE-MRI data	0.39	**0.29**	0.58	0.64	0.65	0.71	0.70	0.78
	Skeletal muscle regeneration gene expression data	0.51	0.71	**0.45**	0.69	0.73	0.74	0.79	0.82
