# Supplementary material for: Convex Analysis of Mixtures for Separating Non-negative Well-grounded Sources
Source: Sci Rep. 2016 Dec 6;6:38350. doi: 10.1038/srep38350 (PMC5138607; doi:10.1038/srep38350)
Supplement: Supplementary Information [file srep38350-s1.pdf]

Convex Analysis of Mixtures for Separating Non-negative Well-grounded Sources (Supplementary Information)

Yitan Zhu[1,2], Niya Wang[1], David J. Miller[3], and Yue Wang[1]

1. The Bradley Department of Electrical and Computer Engineering, Virginia Polytechnic Institute and State University, Arlington, VA 22203, USA

2. The Program of Computational Genomics and Medicine, NorthShore University HealthSystem, Evanston, IL 60201, USA

3. The Department of Electrical Engineering, Pennsylvania State University, University Park, PA 16802, USA

Corresponding Author

Yue Wang

Address: 900 N. Glebe Road, Arlington, VA 22203, USA

Phone: 571-858-3150

Email: yuewang@vt.edu

Contents

1. Brief Review of Existing BSS Methods and Their Relationship to CAM 3
2. Definitions of Recovery Accuracies on Mixing Matrix and Sources 8
3. Performance Comparison With Benchmark BSS Methods on Numerically Mixed Gene Expression Data 9
4. Sensitivity Study of Removing Small-norm Data Points for Data Preprocessing 16
5. Proof of Lemma 1 20
6. Proof of Lemma 2 21
7. Proof of Theorem 2 22
8. Proof of Theorem 3 23
9. References 25

1. Brief Review of Existing BSS Methods and Their Relationship to CAM

Over the past fifteen years, a variety of BSS techniques have been continuously reported and tested on synthetic and real data, including Independent Component Analysis (ICA) and its variants, which assume sources are mutually statistically independent or uncorrelated[1-3], and Non-negative Matrix Factorization (NMF) and its variants, which assume mixing proportions and sources are non-negative[4-6]. NMF is known to have non-unique solutions and can be trapped in a local optimum of its objective function[6]. Efforts, such as the incorporation of sparsity constraint[5-6], have been made to obtain more well-posed problems under the NMF framework[6-9]. Some other extensions of NMF include relaxation on the signs of the matrix factorization. Semi-NMF allows the mixing matrix to have mixed signs and convex-NMF further requires column vectors in $\mathbf{A}$ to be convex combinations of data points in $\mathbf{X}$[10]. While these algorithms can usefully extract interesting patterns from mixture observations, they may prove inaccurate or even incorrect in the face of real-world BSS problems, where their pre-imposed assumptions may not be valid. In particular, many source signals are statistically dependent and may not be globally sparse[11-12].

Alternative BSS techniques exploit Well-Grounded Points (WGPs) in non-negative source patterns, i.e. points with very high values in one source relative to all other sources[7,12-13]. Under the assumption of WGPs, column vectors of the mixing matrix can be estimated by identifying WGPs located at the corners of the mixture observation scatter simplex and, subsequently, the hidden source signals can be recovered. N-FINDR is one of the earliest methods based on WGPs and identifies WGPs by searching for the maximum-volume simplex formed by the data points[14]. Vertex Component Analysis (VCA) implements a fast WGP

detection scheme by iteratively projecting data onto a direction orthogonal to the subspace spanned by the WGPs already determined and selecting the data point corresponding to the most extreme_projection as the next WGP[15]. The maximum-volume strategy has also been applied in the signal space by nonnegative least-correlated component analysis for recovering well-grounded sources[7]. A linear programming method has been used to identify WGPs by examining each observed data point to see whether it is confined within the cone formed by other data points[16]. For cases where WGPs are absent but nearly pure-source data points exist, a constrained NMF method considering both the reconstruction error and the minimization of the simplex volume determined by the estimated mixing matrix column vectors has been proposed[17]. A post-processing framework on the results obtained by a WGP-based solution has been developed using either extra mixture data or reliable peak structures of source signals, also for the situations where WGPs are absent[18].

However, there are several potential limitations associated with existing techniques and our CAM work addresses all these limitations. First, existing methods usually lack a theoretical proof of model identifiability and solution optimality[12]. Many methods adopt the strategy of identifying WGPs without a stringent mathematical framework showing its validity. Second, many existing methods can be used only in the exact-determined and over-determined cases, where the number of mixtures is no less than the number of sources, but not in the under-determined case, where there are more sources than mixtures. Third, their solutions (including model selection) may be sensitive to noise and outliers in the data. Fourth, some methods do not allow negative elements in the mixing matrix, which limits their applicability. Fifth, many methods lack the ability to detect the number of sources and thus require this number to be known *a priori*.

Let us further discuss the difference between CAM and some recent works on separating non-negative well-grounded sources. There is significant prior literature studying the separation of non-negative well-grounded sources within the NMF framework[19-23]. Kumar et al. proposed a method that identifies WGPs one-by-one by detecting the extreme ray farthest away from the cone formed by the WGPs that have already been detected[20]. Esser et al. developed a convex model for NMF that also uses a clustering method to reduce data points and noise[19]. Benson et al. proposed a scalable and efficient method for solving problems where $M \gg N$[21]. Gillis and Luce proposed a linear programming model to robustly identify well-grounded sources from noisy data[22]. Compared to these methods**,** which are based on a non-negative mixing matrix, CAM has greater applicability by allowing the mixing matrix to have both positive and negative elements. Gillis et al. presented a fast recursive algorithm for identifying well-grounded sources which requires the mixing matrix to have full column rank but allows it to have mixed signs[23]. Compared to this work, CAM can identify not only a fully ranked mixing matrix, but also a simplicial mixing matrix with linearly dependent column vectors. Kim and Smaragdis modeled data with multiple small convex cones to accommodate manifold structure in the source signals[24], whereas CAM fits one convex cone to the data.

Compared to our previous works for separating non-negative well-grounded sources, such as nonnegative Least-correlated Component Analysis (nLCA)[7] and Convex Analysis of Mixtures of Non-negative Sources (CAMNS)[13], our current work CAM has several significant differences and advantages. First, CAM operates in the M-dimensional scatter space. It first identifies the mixing matrix through edge detection in the scatter plot, and then recovers the sources using the estimated mixing matrix. CAM requires only a limited number of WGPs or near-WGPs and its power for estimating the model parameters mainly depends on the diversity

of mixing proportions. Methods like nLCA and CAMNS are different. They operate in the N-dimensional signal space, in which they identify a $K-1$ dimensional subspace where the source signal vectors reside. They then estimate the source signal vectors by either identifying the convex hull extreme points or maximizing the volume of the solid region formed by the estimated sources. Second, as a theoretical contribution of our current work, we prove for the first time a sufficient and necessary condition for identifying the mixing matrix through edge detection, which is the assumption ($A3$), i.e. simplicial mixing matrix. Methods like nLCA and CAMNS depend on the assumption that the mixing matrix has full column rank, which is the assumption ($A4$), a sufficient but not necessary condition for ($A3$) to hold. ($A4$) can only be valid in the exact-determined and over-determined scenarios, which limits the applicability of these methods to these two scenarios. But CAM, based on ($A3$), can identify the mixing matrix and source number in all cases -- exact-, over-, and under-determined scenarios. Third, the CAM algorithm includes a model order selection component to identify the source number, while methods like nLCA and CAMNS do not. They require the true source number to be known a priori as an input parameter. Fourth, methods like nLCA and CAMNS use front-end Principal Component Analysis (PCA) to reduce the rank (dimensionality) of the data and assume the number of sources is one greater than the reduced data rank. The CAM algorithm does not require rank or dimensionality reduction. Without prior knowledge or an accurate estimation of the number of sources, rank or dimensionality reduction may over-reduce the data rank or dimensionality, with the grave consequence of transforming an exact-determined or over-determined problem into an under-determined one, for which the sources are usually unidentifiable.

There is also literature discussing NMF identifiability conditions[25-26]. Donoho and

Stodden proposed conditions under which there is a unique NMF solution for separating well-grounded sources[25]. However, the conditions are restrictive as they only apply to sources consisting of a finite set of "parts" and a finite set of articulations of these parts. Uniqueness of the solution is only ensured when the mixtures contain all different combinations of the parts and their articulations, which may be unrealistic in practice. Comparatively, CAM exploits constraints on the mixing matrix to ensure its identifiability, and thus can be applied when there are a limited number of mixtures. Arora et al. proved the existence of an NMF algorithm that runs in polynomial time and outputs well-grounded sources and a non-negative simplicial mixing matrix that considers only $\mathbf{a}_k \notin C\{\mathbf{A}_{-k}\}, \forall k \in \{1,...,K\}$[26]. Our CAM work can be viewed as an extension of their work by proposing in assumption (A3) a simplicial mixing matrix that allows mixed signs to ensure its identifiability.

Both probabilistic methods and deterministic methods have been used to solve BSS problems, and there is usually a connection between the two kinds of methods[27]. The proposed CAM method is largely a deterministic approach. It is an interesting topic to build a probabilistic model for separating non-negative well-grounded sources. We are currently investigating a probabilistic CAM model that combines geometric convex analysis with probabilistic modeling. Within a probabilistic modelling framework, information-theoretic criteria, such as minimum description length[12], can be used for model selection to determine the source number.

2. Definitions of Recovery Accuracies on Mixing Matrix and Sources

We evaluate the algorithm performance by comparing the estimates of the mixing matrix and sources to the ground truth, together with the accuracy of source number estimation measured over a number of data set replications. We apply the minimum average angle to assess the accuracy in estimating the true mixing matrix $\mathbf{A}$, defined as

$$E_{\mathbf{A}} = 1 - \frac{1}{\pi}\angle\left(\mathbf{A}, \widehat{\mathbf{A}}\right),$$

where $\widehat{\mathbf{A}}$ is the estimate of $\mathbf{A}$. $E_{\mathbf{A}}$ takes a value between 0 and 1, with $E_{\mathbf{A}} = 1$ indicating perfect estimation. The calculation of minimum average angle produces an association between the column vectors in $\widehat{\mathbf{A}}$ and the column vectors in $\mathbf{A}$, which also indicates the association between estimated sources and ground truth sources. To assess the accuracy of source recovery, we use the average correlation coefficient between true sources and their estimates, i.e.

$$E_{\mathbf{S}} = \frac{1}{K}\sum_{k=1}^{K} \rho\left(\mathbf{s}^k, \hat{\mathbf{s}}^k\right),$$

where $\hat{\mathbf{s}}^k$ is the estimate of the kth source $\mathbf{s}^k$ that is the kth row of source matrix $\mathbf{S}$, and $\rho(\cdot,\cdot)$ denotes the correlation coefficient between two input vectors.

3. Performance Comparison With Benchmark BSS Methods on Numerically Mixed Gene Expression Data

We compared the performance of CAM with eight most relevant methods, including non-negative Independent Component Analysis (nICA)[2], Statistical Non-negative Independent Component Analysis (SNICA)[3], Non-negative Matrix Factorization (NMF)[4], Sparse Non-negative Matrix Factorization (SNMF)[5], N-finder algorithm (N-FINDR)[14], Vertex Component Analysis (VCA)[15], Convex Analysis of Mixtures of Non-negative Sources (CAMNS)[13], and Nonnegative Least-Correlated Component Analysis (nLCA)[7].

As a more complex problem, we considered numerical mixtures of four real microarray gene expression profiles ($K = 4$), which are from four distinct ovarian cancer subtypes, i.e. serous, mucinous, endometrioid, and clear cell[28]. The sample labels of the gene expression profiles serving as sources are CHTN-OS-115, UM-OM-001, CHTN-OE-047, and CHTN-OC-033[28]. The sources contain expression levels of $N = 7069$ genes, some of which are approximately WGPs. The source profiles are highly correlated, with an average pair-wise correlation coefficient of 0.83; also, the source vectors of many genes have very small vector norms. To enable applicability of the NMF methods, we limited mixing matrices to be non-negative. We consider exact-determined ($M = K = 4$), over-determined ($M = 6 > K = 4$), and under-determined ($M = 3 < K = 4$) scenarios, 100 randomly constructed mixing matrices for each scenario, and 6 different SNR levels based on zero-mean white Gaussian additive noise. The mixing matrices are required to have unit row-sums. In the exact-determined and over-determined scenarios, they have a condition number ≤ 4, so that ($A4$) holds well. In the under-determined scenario, they satisfy that $\forall \mathbf{a}_k \in \{\mathbf{a}_1,...,\mathbf{a}_K\}$, $\angle\left(\mathbf{a}_k,\mathbf{a}'_{k,C\{\mathbf{A}_{-k}\}}\right) \geq \pi/7$ to ensure that ($A3$)

holds well, where $\mathbf{a}'_{k,C\{\mathbf{A}_{-k}\}}$ is the projection of $\mathbf{a}_k$ on $C\{\mathbf{A}_{-k}\}$. To enable the applicability of NMF and SNMF, all observed negative values in data were truncated to 0. In total, there are 1,800 simulation data sets.

For CAM, we set the sector numbers $J = 20$ and $J = 30$, with the results indexed by CAM-20S and CAM-30S, respectively. Data preprocessing removed half of the data points with small vector norms. The sector-based clustering always chose the best outcome from 20 independent runs. Stability analysis used 30 cross-validations. We calculated the performance measures for recovering the mixing matrix and the whole gene expression source profiles. More importantly, we also calculated source recovery accuracy over the top source-specific genes -- 800 genes for each ovarian cancer subtype, selected to maximize $s_{k,n}/\sum_{i=1}^{K} s_{i,n}$, $\forall k = 1,\ldots,4$. The distinct source patterns over these genes that are highly expressed in a specific ovarian cancer subtype are of great interest in biological study[29].

When evaluating the accuracies of recovering the mixing matrix, sources, and distinct patterns of sources, the number of sources ($K = 4$) was assumed known and used as an input parameter for all the algorithms. All mixture gene expression profiles were normalized by scaling to have a unit sum before applying CAM and other methods. Principal Component Analysis (PCA) was used to convert an over-determined case to an exact-determined case when applying nICA, SNICA, and N-FINDR in the over-determined experimental scenario[1,7], because these methods can only work in the exact-determined case. Random initialization was used for setting the initial algorithm parameters needed to run the methods. NMF used the multiplicative update rule proposed in[30]. SNMF used the multiplicative update rule proposed in[5], with the source sparseness and model fitting error equally weighted in its objective function. NMF and SNMF terminated when the absolute changes of their objective function values were no larger

than 0.0001% or when their numbers of interactions exceeded 5000. The iterative gradient search algorithm of nICA terminated when the mean squared error or its absolute change is smaller than 1×10^{-9} or when the number of interactions exceeded 5000[2]. SNICA used a simulated annealing algorithm based on constrained Metropolis-type Monte Carlo search to minimize the mutual information between recovered sources[3]. In the initial stage, the Metropolis temperature parameter was set at 0.01, and in the refine stage it was set at 1×10^{-6}. The algorithm terminated when the minimum mutual information obtained during the entire run did not decrease in 200 successive Monte Carlo steps. The VCA algorithm requires the SNR to either be estimated or to be input to the algorithm. We found that VCA performance was very poor when the algorithm used its own internal estimation of the SNR. Thus, in our experiments we input the SNR as 100 dB, which basically indicates the data is almost noise-free. This gave more reasonable VCA performance. Random initialization was used for CAMNS as suggested in the original paper[13].

Fig. S1 shows the performance results in the exact-determined and over-determined scenarios, when the correct number of sources is given. The estimation accuracies on the mixing matrix, whole hidden sources, and distinct source patterns, are averages over 100 simulation datasets. It can be seen that both CAM-20S and CAM-30S outperform all eight peer methods in all cases, and most importantly, they consistently achieve higher accuracy in recovering the distinct source patterns. It should be noted that the use of an overall correlation coefficient in assessing the estimation accuracy of sources may be misleading when the underlying sources are already highly correlated, and the correlation coefficient calculated over the distinct source patterns should be a more meaningful accuracy measure[12]. It is not surprising to see that the source recovery accuracies of nICA were usually lower than those of other methods, because the sources in this comparison are correlated and violate the basic assumption of nICA that the

sources must be uncorrelated. In all circumstances NMF and SNMF consistently produced similar results, indicating when the sources are not globally and sufficiently sparse, the difference between the performances of SNMF and NMF is insignificant. Though VCA, N-FINDR, nLCA, and CAMNS also exploit the idea of well-grounded sources, they are very sensitive to noise or outliers and thus produce unsatisfactory performance compared to CAM.

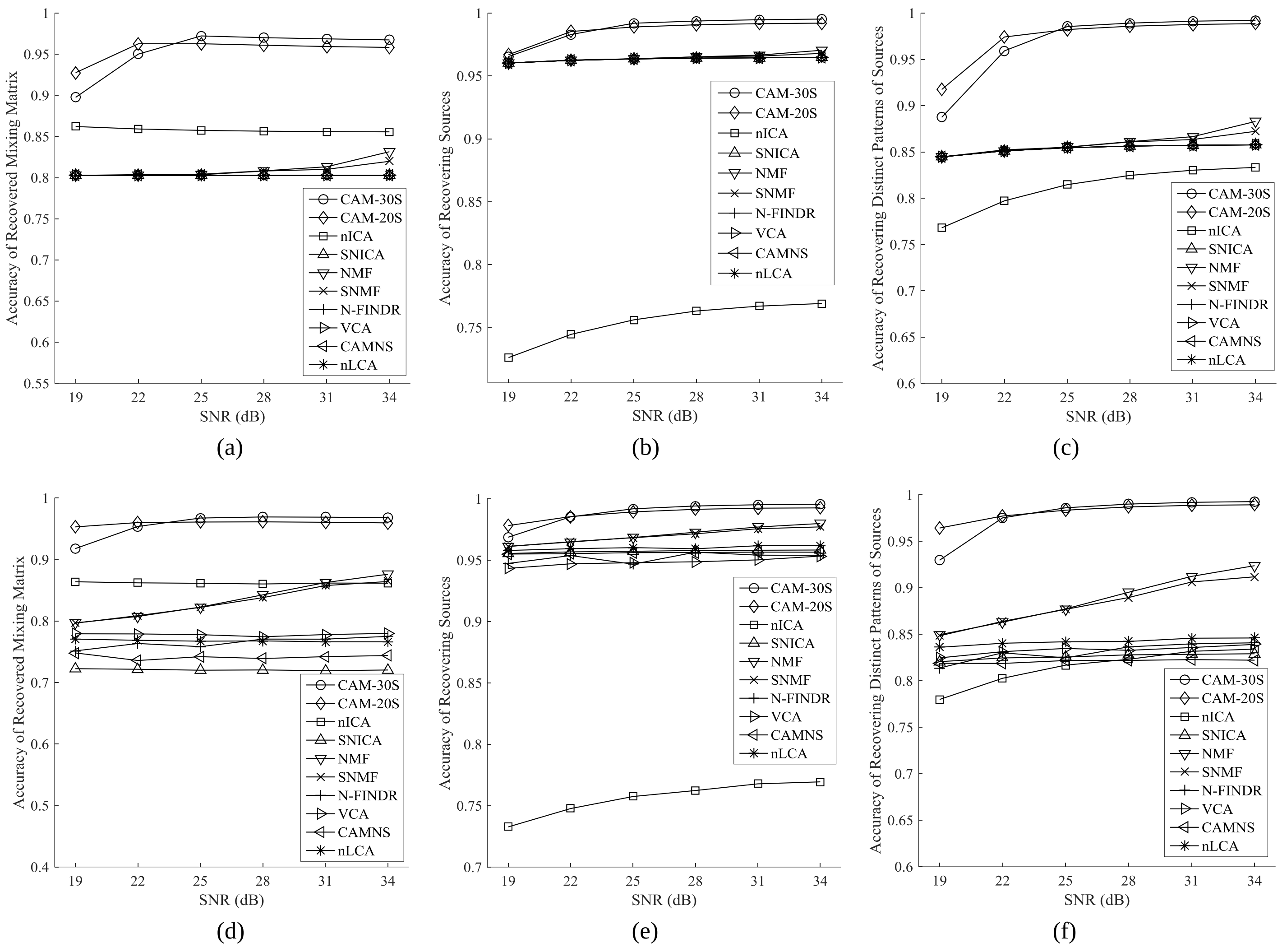

Figure S1 Performance comparison of CAM and peer methods. (a) and (d) are comparisons on accuracy of recovering the mixing matrix in the exact-determined scenario and over-determined scenario, respectively. (b) and (e) are comparisons on accuracy of recovering sources in the exact-determined scenario and over-determined

scenario, respectively. (c) and (f) are comparisons on the accuracy of recovering distinct patterns of sources in the exact-determined scenario and over-determined scenario, respectively.

To assess the performance of stability based model selection (a unique feature of CAM) at each SNR level, we measured the frequency with which CAM correctly detected the number of sources, over the 100 simulation datasets. Fig. S2 shows this accuracy at different SNR levels in exact-determined and over-determined scenarios. For both CAM-30S and CAM-20S, the number of sources ($K = 4$) was always accurately detected for SNR equal to or higher than 25dB in exact-determined and over-determined scenarios. At lower SNR levels, CAM-20S shows a more robust performance against noise than CAM-30S.

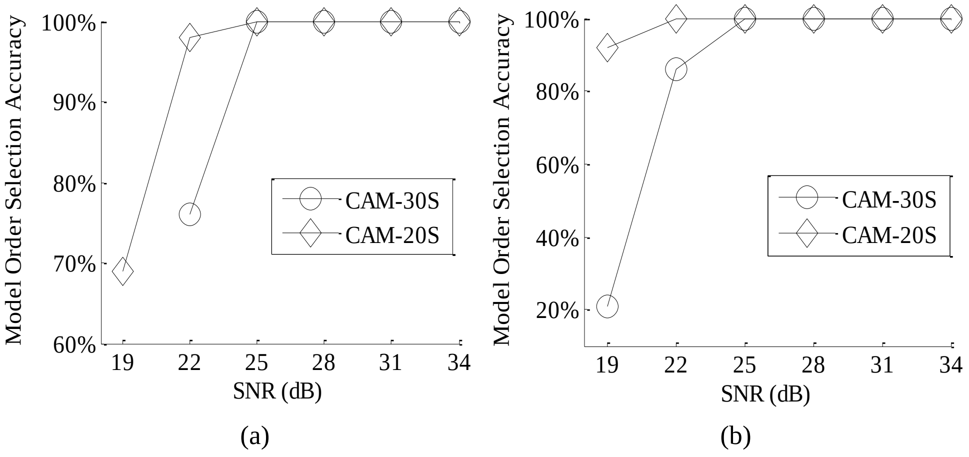

Figure S2 Model order selection accuracy of CAM. (a) and (b) are the model order selection accuracies obtained in the exact-determined and over-determined scenarios, respectively. The model order selection accuracy of CAM-30S at 19dB is 97% and not drawn in (a), because it is misleading. At 19dB, some of the estimates of mixing matrix obtained by CAM-30S tend to be a permutation and scaling matrix, which indicates poor unmixing. Without effective unmixing, the mixture data dimension is mistaken as the estimated source number that equals the true source number in the exact-determined case, which gives rise to the misleading high model order selection accuracy.

Fig. S3a shows that CAM can recover the mixing matrix reasonably well over the entire tested SNR range in the under-determined scenario, when the number of sources is given. Fig. S3b shows the accuracy of model order selection in the under-determined scenario, indicating that when the SNR level is higher than 25dB both CAM-20S and CAM-30S detect the correct source number (i.e. 4) on more than 80% of the datasets. In both Fig. S3a and Fig. S3b, some slight performance drop is observed in the under-determined scenario when the SNR is increased toward its high end, possibly due to over-compensation for the noise by the clustering scheme when the noise level is low.

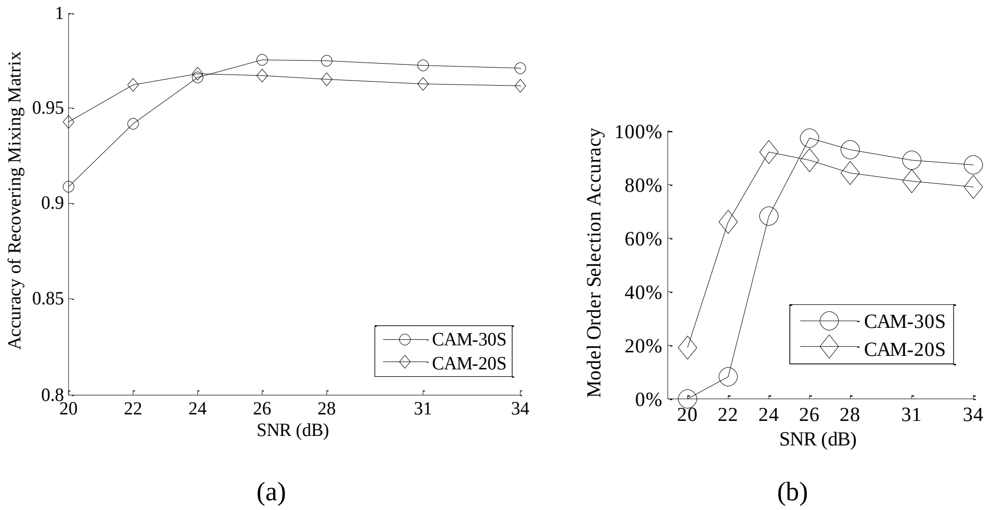

Figure S3 The performance of CAM on recovering (a) the mixing matrix and (b) the source number in the under-determined scenario.

To compare computational complexity of the methods, we recorded the execution times of all methods, analyzing the 100 datasets of 22dB SNR in the exact-determined scenario on a computer with a 1.60GHz CPU. The analyses were run with the true number of sources known and with the parameter setting as described above. All methods were implemented in Matlab for

a fair comparison, expect for SNICA, which was implemented in C. The mean and standard deviation of execution times in seconds are presented in Table S1. VCA is the fastest among all methods, followed by nLCA, and then nICA, CAMNS, SNMF, N-FINDR and NMF. CAM is slower than these methods, but faster than SNICA, which is the slowest among all competing methods, even with its implementation in C. CAM-30S is slower than CAM-20S as expected, because sector-based clustering takes more time when there are more sectors and the estimation of mixing matrix column vectors through minimization of model fitting error may also take more time due to possibly a larger number of detected edges.

Table S1 Comparison of execution times (in seconds) for different methods

Method	Mean	Standard Deviation
CAM-20S	24.45	2.51
CAM-30S	33.00	4.03
NMF	7.87	4.31
nICA	1.59	0.33
N-FINDR	5.66	0.04
SNMF	5.15	1.97
SNICA	62.11	2.31
VCA	0.02	0.01
CAMNS	3.84	2.08
nLCA	1.11	0.04

4. Sensitivity Study of Removing Small-norm Data Points in Data Preprocessing

In the data preprocessing step, a portion of the data points whose norms are small are excluded for the estimation of the mixing matrix, because they potentially have low local SNR. A different percentage of data points, varied from 30% to 50%, were removed in each of the analyses conducted. Here, we study how sensitive the analysis results are to the choice of the percentage of data points removed.

The analysis of breast cancer DCE-MRI data was already performed removing 30% of the pixels whose vector norms were small (see the section of Analysis of Breast Cancer DCE-MRI Data in the main text). We added two more experiments performing the same analysis, but removing 40% and 50% of the pixels whose vector norms are small, while keeping all other algorithm parameters unchanged. Table S2 shows the NMI indices associated with different potential source numbers for model order selection. The optimal source number with the minimum NMI index (indicated by bold font in Table S2) is 3 for both experiments and is the same as the source number detected in the original analysis with 30% of the pixels removed (see Table 1). Fig. S4 shows the tracer concentration changes of the identified compartments over time, which are the mixing matrix column vectors after scaling to have a unit sum. Comparing Fig. S4 with Fig. 4b, we see that both new experiments give time-course tracer concentration changes almost identical to those obtained in the original analysis with 30% of the pixels removed. Also, we use numeric measurements $E_{\mathbf{A}}$ and $E_{\mathbf{S}}$ (defined in Supplementary Information Section 2) to evaluate the similarity between results obtained with different percentages of data points excluded. Both $E_{\mathbf{A}}$ and $E_{\mathbf{S}}$ are between 0 and 1, with 1 indicating $\mathbf{A}$ and $\widehat{\mathbf{A}}$ or $\mathbf{S}$ and $\widehat{\mathbf{S}}$ perfectly match. Taking the mixing matrix and sources estimated with 30% of the pixels

removed as $\mathbf{A}$ and $\mathbf{S}$ and the mixing matrix and sources estimated with 40% of the pixels removed as $\widehat{\mathbf{A}}$ and $\widehat{\mathbf{S}}$, we calculate $E_{\mathbf{A}} = 0.9966$ and $E_{\mathbf{S}} = 0.9996$. Keeping $\mathbf{A}$ and $\mathbf{S}$ unchanged and taking the mixing matrix and sources estimated with 50% of the pixels removed as $\widehat{\mathbf{A}}$ and $\widehat{\mathbf{S}}$, we have $E_{\mathbf{A}} = 0.9955$ and $E_{\mathbf{S}} = 0.9982$. We can see when the percentage of pixels removed is varied between 30% and 50%, CAM outputs highly similar (stable) results, including the estimated mixing matrix and sources.

Table S2 NMI indices associated with different source numbers obtained on the DCE-MRI data.

Source Number	2	3	4	5	6	7	8	9
DCE-MRI data (removing 40% of data points)	0.33	**0.29**	0.56	0.65	0.74	0.67	0.66	0.73
DCE-MRI data (removing 50% of data points)	0.34	**0.32**	0.67	0.54	0.70	0.75	0.74	0.76

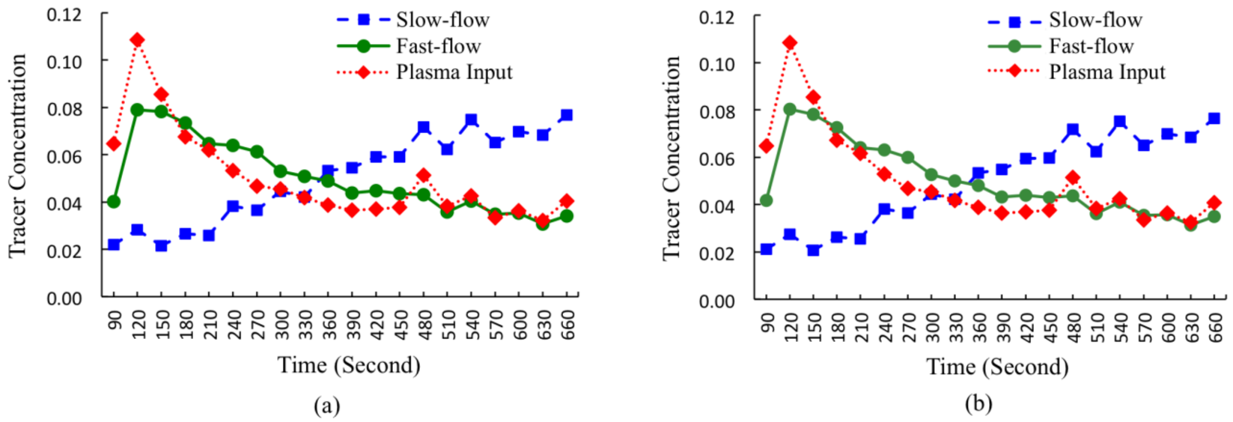

Figure S4 Tracer concentration changes of the identified compartments over time. (a) Analysis results with 40% of the pixels removed. (b) Analysis results with 50% of the pixels removed.

The original analysis of skeletal muscle regeneration gene expression data was performed removing 40% of the genes whose vector norms were small (see the section of Analysis of

Muscle Regeneration Time-Course Gene Expressions in the main text). We added two more experiments performing the same analysis but while removing 30% and 50% of the genes whose vector norms are small, and keeping all other algorithm parameters unchanged. Table S3 shows the NMI indices associated with different potential source numbers for model order selection, The optimal source number with the minimum NMI index (indicated by bold font in Table S3) is 4 for both experiments and is identical to the source number detected in the original analysis with 40% of the genes removed (see Table 1). Fig. S5 shows the time activity curves of the identified sources that are the mixing matrix column vectors. Comparing Fig. S5 with Fig. 5, we can see both new experiments give time activity curves highly similar to those obtained in the original analysis with 40% of the genes removed. Taking the mixing matrix and sources estimated with 40% of the genes removed as **A** and **S** and the mixing matrix and sources estimated with 30% of the genes removed as $\widehat{\mathbf{A}}$ and $\widehat{\mathbf{S}}$, we calculate $E_{\mathbf{A}} = 0.9900$ and $E_{\mathbf{S}} = 0.9988$. Keeping **A** and **S** unchanged and taking the mixing matrix and sources estimated with 50% of the genes removed as $\widehat{\mathbf{A}}$ and $\widehat{\mathbf{S}}$, we have $E_{\mathbf{A}} = 0.9912$. and $E_{\mathbf{S}} = 0.9986$. We can see that when the percentage of removed genes is varied between 30% and 50%, CAM outputs highly similar (stable) results.

The sensitivity study shows that the analysis results of CAM, including the estimated mixing matrix and sources, are quite stable when the percentage of removed small-norm data points changes over a relatively large range, i.e. 30%~50%. A reason for such stable results is that the mixing matrix column vectors are estimated based on sector central rays obtained through sector-based clustering, which relies more on large-norm data points than small-norm data points, because the large-norm data points contribute more to the clustering distortion that the sector-based clustering algorithm locally minimizes.

Table S3 NMI indices associated with different source numbers obtained on skeletal muscle regeneration gene expression data.

Source Number	2	3	4	5	6	7	8	9
Skeletal muscle regeneration gene expression data (removing 30% of data points)	0.54	0.71	**0.43**	0.57	0.64	0.72	0.76	0.77
Skeletal muscle regeneration gene expression data (removing 50% of data points)	0.46	0.68	**0.45**	0.59	0.67	0.67	0.70	0.74

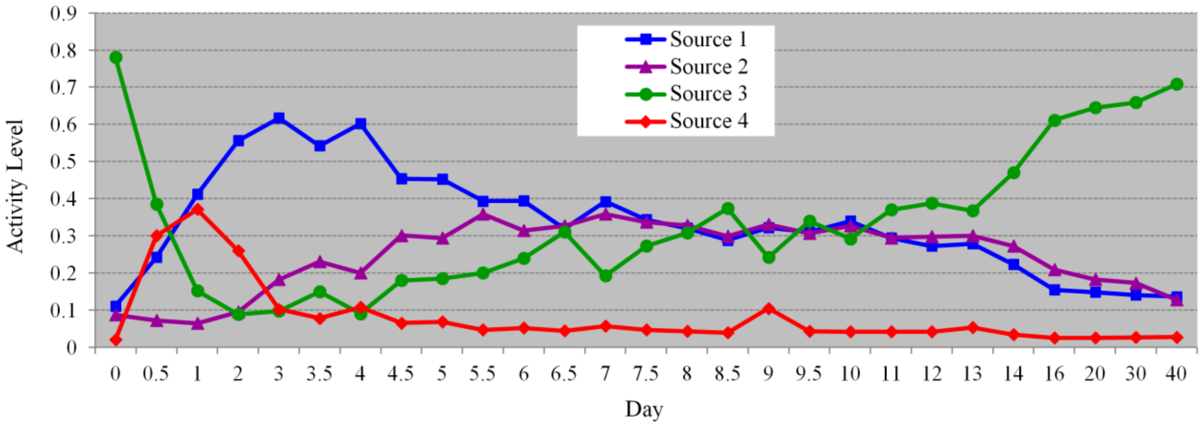

(a)

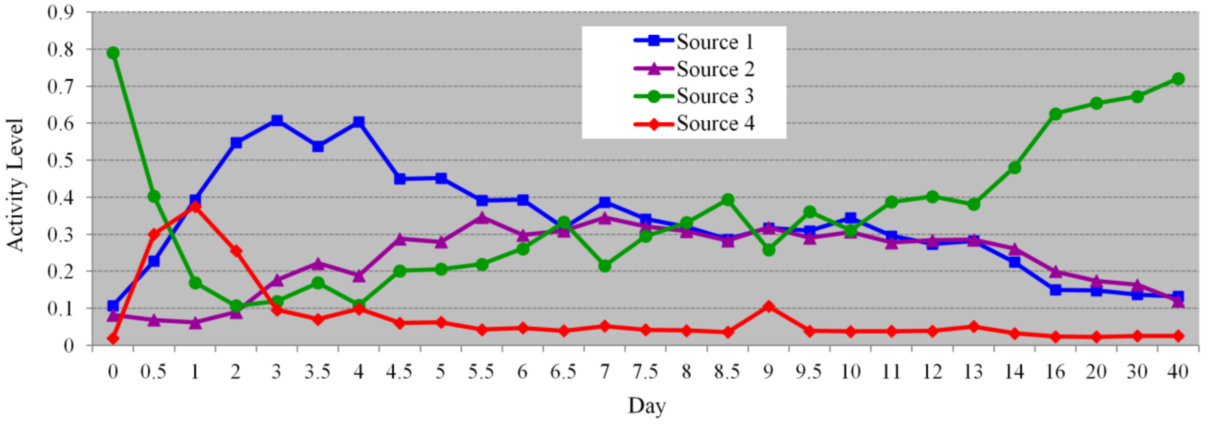

(b)

Figure S5 Time activity curves of the identified sources detected on the skeletal muscle regeneration gene expression data. (a) Analysis results with 30% of the genes removed. (b) Analysis results with 50% of the genes removed.

4. Proof of Lemma 1

First, we prove that $(A3)$ is a sufficient condition. Suppose that $(A3)$ holds. $\forall \mathbf{a}_k \in \{\mathbf{a}_1,...,\mathbf{a}_K\}$, because $\mathbf{a}_k \in C\{\mathbf{A}\}$, $\mathbf{a}_k$ can be represented by $\mathbf{a}_k = \sum_{j=1}^{K} \alpha_j \mathbf{a}_j$, $\alpha_j \geq 0$, $\forall j \in \{1,...,K\}$. Then we have $(1-\alpha_k)\mathbf{a}_k = \sum_{j=1,j\neq k}^{K} \alpha_j \mathbf{a}_j$, which indicates $\alpha_k = 1$, because otherwise $\mathbf{a}_k \in C\{\mathbf{A}_{-k}\}$ or $\mathbf{a}_k \in C\{-\mathbf{A}_{-k}\}$, and $(A3)$ is violated. Thus, $\sum_{j=1,j\neq k}^{K} \alpha_j \mathbf{a}_j = \mathbf{0}$. Because $(A3)$ holds, $\forall j \neq k$, $\mathbf{a}_j \notin C\{-\mathbf{A}_{-(k,j)}\} \subseteq C\{-\mathbf{A}_{-j}\} \Rightarrow \alpha_j = 0$, where $\mathbf{A}_{-(k,j)}$ is the matrix resulting from removing the kth and jth columns from $\mathbf{A}$. This indicates that $\mathbf{a}_k$ must be a trivial non-negative combination of $\{\mathbf{A}\}$ and thus $\mathbf{a}_k$ is an edge.

Second, we prove that $(A3)$ is a necessary condition. Suppose that $(A3)$ is not satisfied. Then, $\exists k \in \{1,\ldots,K\}$, $\mathbf{a}_k \in C\{\mathbf{A}_{-k}\}$ or $\mathbf{a}_k \in C\{-\mathbf{A}_{-k}\}$. Also, $\mathbf{a}_k \neq \gamma \mathbf{a}_j$, $\forall j \in \{1,\ldots,K\}, j \neq k$, and $\forall \gamma > 0$, because otherwise the model is degenerate. $\mathbf{a}_k \in C\{\mathbf{A}_{-k}\} \Rightarrow \mathbf{a}_k = \sum_{j=1,j\neq k}^{K} \alpha_j \mathbf{a}_j$, $\alpha_j \geq 0$, $\forall j \in \{1,\ldots,K\}$ and $j \neq k$. Because $\mathbf{a}_k \neq \mathbf{0}$, $\exists \alpha_j > 0$ and $j \neq k$. We can represent $\mathbf{a}_k$ by $\mathbf{a}_k = \mathbf{a}_k/2 + \sum_{j=1,j\neq k}^{K} (\alpha_j/2)\mathbf{a}_j$. Thus $\mathbf{a}_k$ is a non-trivial combination of $\{\mathbf{A}\}$ and is not an edge. In a similar way, we can show that $\mathbf{a}_k \in C\{-\mathbf{A}_{-k}\}$ also makes $\mathbf{a}_k$ a non-trivial combination of $\{\mathbf{A}\}$ and thus is not an edge. This indicates that $(A3)$ must be satisfied for $\{\mathbf{a}_1,...,\mathbf{a}_K\}$ to be the edges.

5. Proof of Lemma 2

Any vector $\mathbf{v} \in C\{\mathbf{X}\}$ can be represented by $\mathbf{v} = \sum_{n=1}^{N} \alpha_n \mathbf{x}_n = \sum_{n=1}^{N} \alpha_n \mathbf{A}\mathbf{s}_n = \mathbf{A} \sum_{n=1}^{N} \alpha_n \mathbf{s}_n$, where $\alpha_n \geq 0$, $\forall n \in \{1,\ldots,N\}$. Moreover, $\sum_{n=1}^{N} \alpha_n \mathbf{s}_n$ is a K dimensional non-negative vector. Therefore, $\mathbf{v} \in C\{\mathbf{A}\}$, and we have proved $C\{\mathbf{X}\} \subseteq C\{\mathbf{A}\}$.

Let $\{n_{\mathrm{WGP}(1)},\ldots,n_{\mathrm{WGP}(K)}\}$ be the indices of a WGP set, where $\mathbf{x}_{n_{\mathrm{WGP}(k)}}$ is a WGP of source k. Any vector $\mathbf{v} \in C\{\mathbf{A}\}$ can be represented by

$$\mathbf{v} = \sum_{k=1}^{K} \alpha_k \mathbf{a}_k = \sum_{k=1}^{K} \frac{\alpha_k}{s_{k,n_{\mathrm{WGP}(k)}}} \mathbf{x}_{n_{\mathrm{WGP}(k)}},$$

where $\alpha_k \geq 0$, $\forall k \in \{1,\ldots,K\}$. Obviously, $\alpha_k / s_{k,n_{\mathrm{WGP}(k)}} \geq 0$, so $\mathbf{v} \in C\{\mathbf{X}\}$, and we have proved $C\{\mathbf{A}\} \subseteq C\{\mathbf{X}\}$.

Therefore, $C\{\mathbf{A}\} = C\{\mathbf{X}\}$.

6. Proof of Theorem 2

First, we assume that $\angle\left(\mathbf{x}_n,\mathbf{x}'_{n,C\{\mathbf{X}_{-n}\}}\right) > 0$, which means $\mathbf{x}_n \notin C\{\mathbf{X}_{-n}\}$. Because $\mathbf{x}_n \in C\{\mathbf{X}\}$, we can write $\mathbf{x}_n = \sum_{i=1}^{N} \alpha_i \mathbf{x}_i \Leftrightarrow (1-\alpha_n)\mathbf{x}_n = \sum_{i=1,i\neq n}^{N} \alpha_i \mathbf{x}_i$, where $\alpha_i \geq 0$, $\forall i \in \{1,\ldots,N\}$. Because $\mathbf{x}_n \notin C\{\mathbf{X}_{-n}\}$, $\alpha_n \geq 1$. We can further write $\mathbf{0} = \sum_{i=1,i\neq n}^{N} \alpha_i \mathbf{x}_i + (\alpha_n - 1)\mathbf{x}_n = \mathbf{A}\left(\sum_{i=1,i\neq n}^{N} \alpha_i \mathbf{s}_i + (\alpha_n - 1)\mathbf{s}_n\right)$, where $\sum_{i=1,i\neq n}^{N} \alpha_i \mathbf{s}_i + (\alpha_n - 1)\mathbf{s}_n$ is a non-negative vector. Actually, it must be a zero vector, because otherwise ($A3$) is violated. Because ($A1$) is satisfied, $\mathbf{s}_i$ is a non-negative, non-zero vector, $\forall i \in \{1,\ldots,N\}$. Then we must have $\alpha_i = 0$, $\forall i \neq n$, and $\alpha_n = 1$. So $\mathbf{x}_n$ can only be a trivial non-negative combination of $\mathbf{x}_1,\ldots,\mathbf{x}_N$, which means that $\mathbf{x}_n$ is a lateral edge of $C\{\mathbf{X}\}$.

Second, suppose that $\angle\left(\mathbf{x}_n,\mathbf{x}'_{n,C\{\mathbf{X}_{-n}\}}\right) = 0$, which means $\mathbf{x}_n \in C\{\mathbf{X}_{-n}\}$. Also, for simplicity of discussion, assume that $\mathbf{x}_1,\ldots,\mathbf{x}_N$ have different vector directions, i.e. no vector is a positive scaling of another vector. $\mathbf{x}_n$ can be represented by $\mathbf{x}_n = \sum_{i=1,i\neq n}^{N} \alpha_i \mathbf{x}_i$, where $\alpha_i \geq 0$, $\forall i \neq n$, and $\alpha_i > 0$ for at least two data points other than $\mathbf{x}_n$. Thus we can write $\mathbf{x}_n$ as a non-trivial non-negative combination of $\mathbf{x}_1,\ldots,\mathbf{x}_N$, for example, $\mathbf{x}_n = \mathbf{x}_n/2 + \sum_{i=1,i\neq n}^{N} (\alpha_i/2)\mathbf{x}_i$, which means that $\mathbf{x}_n$ is not a lateral edge of $C\{\mathbf{X}\}$. Therefore, $\mathbf{x}_n$ can be a lateral edge of $C\{\mathbf{X}\}$ only if $\angle\left(\mathbf{x}_n,\mathbf{x}'_{n,C\{\mathbf{X}_{-n}\}}\right) > 0$.

7. Proof of Theorem 3

We define maximum source dominance with respect to a normalized version of the data points. Because $\mathbf{A}$ has full column rank, there exist K linearly independent rows in $\mathbf{A}$ forming a basis, i.e. any real-valued K-dimensional vector can be formed by linear combination of the K row vectors in $\mathbf{A}$, including any non-negative vectors. Thus, there must exist a vector $\boldsymbol{\gamma}$ satisfying $\boldsymbol{\gamma}^T\mathbf{a}_k > 0$, $\forall k = 1,...,K$. The mixture data vectors are scaled to have unit inner products with $\boldsymbol{\gamma}$, i.e.

$$\tilde{\mathbf{x}}_n = \frac{\mathbf{x}_n}{\boldsymbol{\gamma}^T\mathbf{x}_n} = \frac{\mathbf{A}\mathbf{s}_n}{\boldsymbol{\gamma}^T\mathbf{x}_n} = \left[\frac{\mathbf{a}_1}{\boldsymbol{\gamma}^T\mathbf{a}_1},\ldots,\frac{\mathbf{a}_K}{\boldsymbol{\gamma}^T\mathbf{a}_K}\right]\begin{bmatrix}\frac{\boldsymbol{\gamma}^T\mathbf{a}_1 s_{1,n}}{\boldsymbol{\gamma}^T\mathbf{x}_n}\\ \vdots \\ \frac{\boldsymbol{\gamma}^T\mathbf{a}_K s_{K,n}}{\boldsymbol{\gamma}^T\mathbf{x}_n}\end{bmatrix} = \widetilde{\mathbf{A}}\tilde{\mathbf{s}}_n,$$

where $\tilde{\mathbf{s}}_n = \left[\boldsymbol{\gamma}^T\mathbf{a}_1 s_{1,n}/\boldsymbol{\gamma}^T\mathbf{x}_n \ldots \boldsymbol{\gamma}^T\mathbf{a}_K s_{K,n}/\boldsymbol{\gamma}^T\mathbf{x}_n\right]^T$ and $\widetilde{\mathbf{A}} = [\mathbf{a}_1/\boldsymbol{\gamma}^T\mathbf{a}_1,\ldots,\mathbf{a}_K/\boldsymbol{\gamma}^T\mathbf{a}_K]$. Obviously, $\tilde{s}_{k,n} = \boldsymbol{\gamma}^T\mathbf{a}_k s_{k,n}/\boldsymbol{\gamma}^T\mathbf{x}_n \geq 0$, $\forall k \in \{1,...,K\}$, and $\sum_{k=1}^{K}\tilde{s}_{k,n} = 1$. $\tilde{s}_{k,n}$ defines the level/abundance of source k in the nth data point after normalization. Because the normalization only performs a positive scaling of the data vectors, the lateral edges of $C\{\widetilde{\mathbf{X}}\}$ remain the same as those of $C\{\mathbf{X}\}$ and thus can be identified by the edge detection strategy implied by Theorem 2.

Consider $\tilde{\mathbf{x}}_{n_k^*}$, whose kth source abundance is the largest, i.e. such that $n_k^* = \operatorname{argmax}_{n=1,\ldots,N}\tilde{s}_{k,n}$,

$$\tilde{\mathbf{x}}_{n_k^*} = \frac{\mathbf{x}_{n_k^*}}{\boldsymbol{\gamma}^T\mathbf{x}_{n_k^*}} = \frac{\sum_{n=1}^{N}\alpha_n\mathbf{x}_n}{\boldsymbol{\gamma}^T\sum_{n=1}^{N}\alpha_n\mathbf{x}_n} = \sum_{n=1}^{N}\frac{\alpha_n\boldsymbol{\gamma}^T\mathbf{x}_n}{\sum_{i=1}^{N}\alpha_i\boldsymbol{\gamma}^T\mathbf{x}_i}\frac{\mathbf{x}_n}{\boldsymbol{\gamma}^T\mathbf{x}_n} = \sum_{n=1}^{N}\tilde{\alpha}_n\tilde{\mathbf{x}}_n,$$

where $\alpha_n \geq 0$, $\forall n \in \{1,...,N\}$, and $\tilde{\alpha}_n = \alpha_n\boldsymbol{\gamma}^T\mathbf{x}_n/\sum_{i=1}^{N}\alpha_i\boldsymbol{\gamma}^T\mathbf{x}_i$. Obviously, $\tilde{\alpha}_n \geq 0$ and $\sum_{n=1}^{N}\tilde{\alpha}_n = 1$. Because $\tilde{\mathbf{x}}_{n_k^*} = \sum_{n=1}^{N}\tilde{\alpha}_n\tilde{\mathbf{x}}_n$, we can write

$$\tilde{\mathbf{x}}_{n_k^*} = \sum_{j=1}^{K}\tilde{\mathbf{a}}_j\tilde{s}_{j,n_k^*} = \sum_{n=1}^{N}\tilde{\alpha}_n\sum_{j=1}^{K}\tilde{\mathbf{a}}_j\tilde{s}_{j,n} \Leftrightarrow \sum_{j=1}^{K}\tilde{\mathbf{a}}_j\left(\tilde{s}_{j,n_k^*} - \sum_{n=1}^{N}\tilde{\alpha}_n\tilde{s}_{j,n}\right) = 0.$$

Because $\tilde{\mathbf{a}}_1,\ldots,\tilde{\mathbf{a}}_K$ are linearly independent, $\tilde{s}_{k,n_k^*} - \sum_{n=1}^{N} \tilde{\alpha}_n \tilde{s}_{k,n} = \sum_{n=1}^{N} \tilde{\alpha}_n \left(\tilde{s}_{k,n_k^*} - \tilde{s}_{k,n}\right) = 0$. Define $\Delta_< = \left\{n \middle| \tilde{s}_{k,n} < \tilde{s}_{k,n_k^*}\right\}$ and $\Delta_= = \left\{n \middle| \tilde{s}_{k,n} = \tilde{s}_{k,n_k^*}\right\}$. We have $\tilde{\alpha}_n = 0, \forall n \in \Delta_<$, and thus $\sum_{n\in\Delta_=} \tilde{\alpha}_n = 1$. So, $\tilde{\mathbf{x}}_{n_k^*}$ lies within a convex hull formed by $\{\tilde{\mathbf{x}}_n \,|n \in \Delta_=\}$. Consider a vertex of this convex hull, denoted by $\tilde{\mathbf{x}}_{n_k^{**}}$, which also achieves the maximum dominance of source k. Because $\tilde{\mathbf{x}}_{n_k^{**}} \in C\{\tilde{\mathbf{X}}\}$ and based on the above derivation, we must have $\tilde{\mathbf{x}}_{n_k^{**}} = \sum_{n=1}^{N} \tilde{\beta}_n \tilde{\mathbf{x}}_n, \tilde{\beta}_n \geq 0, \sum_{n\in\Delta_=} \tilde{\beta}_n = 1$, and $\tilde{\beta}_n = 0, \forall n \in \Delta_<$. Because $\tilde{\mathbf{x}}_{n_k^{**}}$ is a vertex of the convex hull, it can only be a trivial combination of $\{\tilde{\mathbf{x}}_n \,|n \in \Delta_=\}$ (i.e. if $\tilde{\beta}_n > 0$ for any $n \in \Delta_=$, then $\tilde{\mathbf{x}}_{n_k^{**}} = \tilde{\mathbf{x}}_n$), which indicates that $\tilde{\mathbf{x}}_{n_k^{**}}$ can only be a trivial combination of $\{\tilde{\mathbf{X}}\}$. Thus $\tilde{\mathbf{x}}_{n_k^{**}}$ is a lateral edge of $C\{\tilde{\mathbf{X}}\}$ and $C\{\mathbf{X}\}$, and can be identified by the CAM solution. Note that $\Delta_= = \{n_k^*\}$ is a special case, wherein the convex hull reduces to a single point vertex, and in such a case $\tilde{\mathbf{x}}_{n_k^*}$ is a lateral edge identified by the CAM solution.

8. References

1 Hyvärinen, A., Karhunen, J. & Oja, E. *Independent Component Analysis* 1edn, (Wiley-Interscience, 2001).

2 Oja, E. & Plumbley, M. Blind separation of positive sources by globally convergent gradient search. *Neural Comput.* **16**, 1811-1825 (2004).

3 Astakhov, S. A., Stögbauer, H., Kraskov, A. & Grassberger, P. Monte carlo algorithm for least dependent non-negative mixture decomposition. *Anal. Chem.* **78**, 1620-1627 (2006).

4 Lee, D. D. & Seung, H. S. Learning the parts of objects by non-negative matrix factorization. *Nature* **401**, 788-791 (1999).

5 Liu, W., Zheng, N. & Lu, X. in *Proc. IEEE Int. Conf. Acoust. Speech Signal Process.*

6 Gillis, N. Sparse and unique nonnegative matrix factorization through data preprocessing. *J. Mach. Learn. Res.* **13**, 3349-3386 (2012).

7 Wang, F. Y., Chi, C. Y., Chan, T. H. & Wang, Y. Nonnegative least-correlated component analysis for separation of dependent sources by volume maximization. *IEEE Trans. Pattern Anal. Mach. Intell.* **32**, 875-888, doi:10.1109/TPAMI.2009.72 (2010).

8 Zhou, G., Xie, S., Yang, Z., Yang, J.-M. & He, Z. Minimum-volume-constrained nonnegative matrix factorization: enhanced ability of learning parts. *IEEE Trans. on Neural Netw.* **22**, 1626–1637 (2011).

9 Huck, A., Guillaume, M. & Blanc-Talon, J. Minimum dispersion constrained nonnegative matrix factorization to unmix hyperspectral data. *IEEE Trans. Geosci. Remote* **48**, 2590–2602 (2010).

10 Ding, C., Li, T. & Jordan, M. I. Convex and Semi-Nonnegative Matrix Factorizations. *IEEE Trans. Pattern Anal. Mach. Intell.* **32**, 45-55 (2010).

11 Hillman, E. M. C. & Moore, A. All-optical anatomical co-registration for molecular imaging of small animals using dynamic contrast. *Nat. Photonics* **1**, 526-530 (2007).

12 Chen, L. *et al.* Tissue-specific compartmental analysis for dynamic contrast-enhanced MR imaging of complex tumors. *IEEE Trans. Med. Imaging* **30**, 2044-2058, doi:10.1109/TMI.2011.2160276 (2011).

13 Chan, T.-H., Ma, W.-K., Chi, C.-Y. & Wang, Y. A convex analysis framework for blind separation of non-negative sources. *IEEE Trans. Signal Proces.* **56**, 5120-5134 (2008).

14 Winter, M. E. in *SPIE Conf. Imaging Spectrometry V.* 266-275.

15 Nascimento, J. M. P. & Dias, J. M. B. Vertex component analysis: a fast algorithm to unmix hyperspectral data. *IEEE Trans. Geosci. Remote* **43**, 898-910 (2005).

16 Naanaa, W. & Nuzillard, J.-M. Blind source separation of positive and partially correlated data. *Signal Process.* **85**, 1711-1722 (2005).

17 Miao, L. & Qi, H. Endmember extraction from highly mixed data using minimum volume constrained nonnegative matrix factorization. *IEEE Trans. Geosci. Remote* **45**, 765-777 (2007).

18 Sun, Y., Ridge, C., Rio, F. d., Shaka, A. J. & Xin, J. Postprocessing and sparse blind source separation of positive and partially overlapped data. *Signal Process.* **91**, 1838–1851 (2011).

19 Esser, E., Möller, M., Osher, S., Sapiro, G. & Xin, J. A convex model for nonnegative matrix factorization and dimensionality reduction on physical space. *IEEE Trans. Image Process.* **21**, 3239-3252 (2012).

20 Kumar, A., Sindhwani, V. & Kambadur, P. in *the 30th International Conference on Machine Learning.*

21 Benson, A. R., Lee, J. D., Rajwa, B. & Gleich, D. F. in *Advances in Neural Information Processing Systems.*

22 Gillis, N. & Luce, R. Robust Near-Separable Nonnegative Matrix Factorization Using Linear Optimization. *J. Mach. Learn. Res.* **15**, 1249-1280 (2014).

23 Gillis, N. & Vavasis, S. A. Fast and Robust Recursive Algorithms for Separable Nonnegative Matrix Factorization. *IEEE Trans. Pattern Anal. Mach. Intell.* **36**, 698-714 (2014).

24 Kim, M. & Smaragdis, P. Mixtures of local dictionaries for unsupervised speech enhancement. *IEEE Signal Processing Letters* **22**, 293-297 (2015).

25 Donoho, D. & Stodden, V. When Does Non-Negative Matrix Factorization Give a Correct Decomposition into Parts? *Proc. Adv. Neural Inf. Process. Syst. 16* (2003).

26 Arora, S., Ge, R., Kannan, R. & Moitra, A. Computing a Nonnegative Matrix Factorization - Provably. *Proc. 44th Symp. Theor. Comput.*, 145-162 (2012).

27 Ding, C., Li, T. & Peng, W. On the equivalence between non-negative matrix factorization and probabilistic latent semantic indexing. *Computational Statistics and Data Analysis* **52**, 3913-3927 (2008).

28 Schwartz, D. R. *et al.* Gene expression in ovarian cancer reflects both morphology and biological behavior, distinguishing clear cell from other poor-prognosis ovarian carcinomas. *Cancer Res.* **62**, 4722-4729 (2002).

29 Yu, G. *et al.* Matched gene selection and committee classifier for molecular classification of heterogeneous diseases. *J. Mach. Learn. Res.* **11**, 2141-2167 (2010).

30 Lee, D. D. & Seung, H. S. in *NIPS.* 556–562.
